# Evaluation of Strategies to Enhance Community-Based Naloxone Distribution Supported by an Opioid Settlement

**DOI:** 10.1001/jamanetworkopen.2024.13861

**Published:** 2024-05-30

**Authors:** Xiao Zang, Alexandra Skinner, Maxwell S. Krieger, Czarina N. Behrends, Ju N. Park, Traci C. Green, Alexander Y. Walley, Jake R. Morgan, Benjamin P. Linas, Jesse L. Yedinak, Bruce R. Schackman, Brandon D. L. Marshall

**Affiliations:** 1 Division of Health Policy and Management, School of Public Health, University of Minnesota, Minneapolis; 2 Department of Epidemiology, School of Public Health, Brown University, Providence, Rhode Island; 3 Department of Population Health Sciences, Weill Cornell Medical College, New York City, New York; 4 Department of Medicine, Brown University, Providence, Rhode Island; 5 The Heller School for Social Policy and Management, Brandeis University, Waltham, Massachusetts; 6 Department of Medicine, Section of General Internal Medicine, Boston Medical Center and Boston University School of Medicine, Boston, Massachusetts; 7 Department of Health Law, Policy & Management, Boston University School of Public Health, Boston, Massachusetts; 8 Section of Infectious Diseases, Boston Medical Center, Boston, Massachusetts; 9 Department of Medicine, Boston University School of Medicine, Boston, Massachusetts

## Abstract

**Importance:**

Many US states are substantially increasing community-based naloxone distribution, supported in part through settlements from opioid manufacturers and distributors.

**Objectives:**

To evaluate the potential impact of increased naloxone availability on opioid overdose deaths (OODs) and explore strategies to enhance this impact by integrating interventions to address solitary drug use.

**Design, Setting, and Participants:**

This decision analytical modeling study used PROFOUND (Prevention and Rescue of Fentanyl and Other Opioid Overdoses Using Optimized Naloxone Distribution Strategies), a previously published simulation model, to forecast annual OODs between January 2023 and December 2025. The simulated study population included individuals from Rhode Island who misused opioids and stimulants and were at risk for opioid overdose.

**Exposures:**

The study modeled expanded naloxone distribution supported by the state’s opioid settlement (50 000 naloxone nasal spray kits each year). Two approaches to expanding naloxone distribution were evaluated: one based on historical spatial patterns of naloxone distribution (supply-based approach) and one based on the spatial distribution of individuals at risk (demand-based approach). In addition, hypothetical interventions to enhance the likelihood of witnessed overdoses in private or semiprivate settings were considered.

**Main Outcomes and Measures:**

Annual number of OODs and ratio of fatal to nonfatal opioid overdoses.

**Results:**

Modeling results indicated that distributing more naloxone supported by the state’s opioid settlement could reduce OODs by 6.3% (95% simulation interval [SI], 0.3%-13.7%) and 8.8% (95% SI, 1.8%-17.5%) in 2025 with the supply-based and demand-based approaches, respectively. However, increasing witnessed overdoses by 20% to 60% demonstrated greater potential for reducing OODs, ranging from 8.5% (95% SI, 0.0%-20.3%) to 24.1% (95% SI, 8.6%-39.3%). Notably, synergistic associations were observed when combining both interventions: increased naloxone distribution with the 2 approaches and a 60% increase in witnessed overdoses could reduce OODs in 2025 by 33.5% (95% SI, 17.1%-50.4%) and 37.4% (95% SI, 19.6%-56.3%), respectively.

**Conclusions and Relevance:**

These findings suggest that interventions to address solitary drug use are needed to maximize the impact of continued efforts to increase community-based naloxone distribution, which may be particularly important for jurisdictions that have strong community-based naloxone distribution programs.

## Key Points

**Question:**

What is the potential impact of the expanded naloxone distribution supported by an opioid settlement have, and how can we enhance this impact by increasing witnessed overdoses?

**Findings:**

In this decision analytical model including a simulated population of Rhode Island residents at risk for opioid overdose, distributing more naloxone supported by the state’s opioid settlement was projected to reduce annual opioid overdose deaths (OODs) by up to 9% with an optimized distribution. The addition of a 60% increase in witnessed overdoses could further reduce annual OODs by 37%.

**Meaning:**

Combining expanded naloxone distribution with efforts to increase witnessed overdoses could result in a greater reduction in OODs than when these 2 strategies are implemented separately.

## Teaser

This decision analytical model evaluates the potential of expanded naloxone distribution supported by an opioid settlement to reduce overdose deaths and assesses strategies to enhance this potential impact with interventions to increase witnessed overdoses.

## Introduction

Opioid overdose mortality in the US has increased substantially in recent years and escalated during the COVID-19 pandemic.^1^ Drug overdose deaths increased by 30% from 2019 to 2020 and by 15% from 2020 to 2021.^2^ The state of Rhode Island faces a particularly high opioid overdose mortality rate of 35.9 per 100 000 population (age adjusted) compared with a national average of 24.7 per 100 000 population in 2021.^3^

An increasingly potent drug supply, largely consisting of synthetic opioids such as fentanyl, continues to drive this spike in fatal overdoses.^4^ Timely administration of naloxone can reverse opioid overdose symptoms, preventing these incidents from becoming fatal.^5^ Widespread naloxone distribution thus has the potential to play a major role in addressing the worsening opioid overdose epidemic in Rhode Island and across the US.^6^ In this context, the primary goal of overdose education and naloxone distribution (OEND) and other community-based naloxone distribution programs is to maximize the availability of naloxone to potential bystanders of an overdose as a harm reduction approach to reduce opioid overdose fatalities.

In a previously published modeling study,^7^ we evaluated the effects of strategies to distribute 10 000 additional naloxone kits each year in Rhode Island (compared with historical distribution rates) and found that as much as 25% of witnessed opioid overdose deaths (OODs) could be averted by further increasing naloxone access among people who inject drugs. However, this assessment was conducted using data before 2020 and accounted for neither the increase in fatal opioid overdoses during the COVID-19 pandemic period nor major efforts to expand naloxone distribution in Rhode Island, which resulted in a quadrupling of the annual number of kits distributed through community-based programs from 2019 to 2022.^8^

To combine and resolve all local and state opioid litigation against a group of opioid manufacturers, distributors, and pharmacies, various legal settlements have been reached nationally that total more than $50 billion.^9^ Among these settlements, Teva Pharmaceuticals agreed to a $4.25 billion settlement that includes the provision of naloxone (in lieu of financial payments) to Rhode Island and other US states that have confirmed participation.^10^ As part of the state’s $20 million settlement with Teva Pharmaceuticals, Rhode Island will receive 50 000 kits containing 2 doses of 4-mg/0.1-mL naloxone intranasal spray annually for 10 years beginning in January 2023.^11^

Although low-barrier access to naloxone is essential for preventing fatal opioid overdose, naloxone is only effective when someone is present to administer it. Unfortunately, only one-third of fatal overdoses in the US occurred with a bystander present, defined as someone being physically present during or shortly preceding a drug overdose who potentially had the opportunity to intervene and respond.^12^ Some recent interventions to address solitary drug use—and thus increase the likelihood that naloxone is administered in the event of an overdose—include peer support programs,^13^ overdose prevention centers (OPCs, sometimes referred to as supervised consumption sites, which are places where people can consume preobtained drugs in a monitored setting where staff can immediately intervene in the event of an overdose),^14^ telephone hotlines, mobile apps, stationary wired or wireless devices (such as passive motion detection system and button alert system), and wearable biosensors.^15^ In addition to expanding naloxone distribution, statewide efforts are under way in Rhode Island to promote and fund some of these initiatives using the state’s opioid settlement funds.^11^

Efforts to reduce solitary drug use may help enhance the effectiveness of naloxone distribution. However, to our knowledge, the potential synergistic effects of expanding naloxone distribution and interventions to reduce solitary drug use have not been examined. Simulation modeling offers a powerful decision tool to synthesize evidence from various sources to evaluate the interventions used in combination, such as naloxone distribution and programs to reduce solitary drug use. The objective of this simulation modeling study is to evaluate the potential impact of the expanded naloxone distribution on OOD supported by the opioid settlement in Rhode Island and how interventions to reduce solitary opioid use could enhance its impact.

## Methods

We adapted a previously calibrated individual-based microsimulation model with an integrated decision tree algorithm, PROFOUND (Prevention and Rescue of Fentanyl and Other Opioid Overdoses Using Optimized Naloxone Distribution Strategies),^7^ to assess the potential outcomes in Rhode Island of distributing 50 000 naloxone nasal spray kits each year supported by the opioid settlement in combination with interventions aiming at reducing solitary drug use. The work was deemed to not be human participants research by the Brown University and Boston University Medical Campus institutional review boards, and therefore no informed consent was required. This decision analytical model followed the Consolidated Health Economic Evaluation Reporting Standards (CHEERS) reporting guideline.

### Model Description

**Figure 1.**
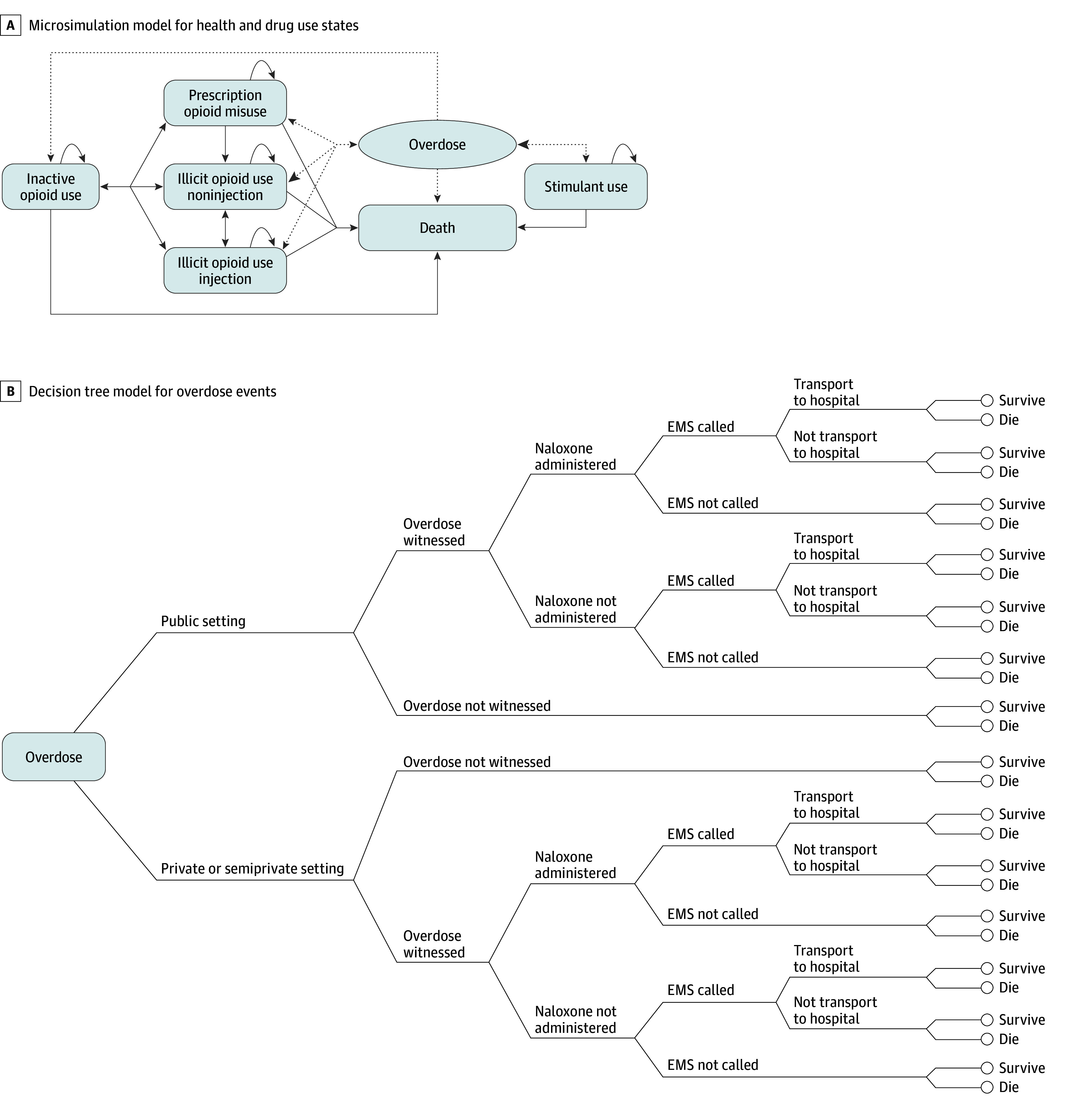
Model Structure Diagram A, Microsimulation model. The oval “Overdose” state represents a tunnel state; solid lines, transitions among health states; dotted lines, transitions between health states and overdose; and arrows, possible directions for state transitions. B, Decision tree model. We defined the overdose settings according to prior studies by the Rhode Island Department of Health,^18,19^ where private settings include apartment or residence; semiprivate settings include hotel, motel, shelter, nursing home, hospital, prison, group home, assisted living, or treatment facility; and public settings include theater, concert, show, office, park, school, bar or restaurant, roadway, or cemetery. EMS indicates emergency medical services.

On model initialization, we created a virtual cohort representing all individuals in Rhode Island who are at risk for opioid overdose using data from the National Survey on Drug Use and Health^16^ and the US census.^17^ The simulated persons were characterized and stratified by sex (male or female), age, race and ethnicity (Black or African American, Hispanic or Latino, non-Hispanic White, or other [any racial or ethnic category that was not Hispanic or Latino, non-Hispanic Black, or non-Hispanic White]), city or town of residence (eFigure 2 in Supplement 1), patterns of drug use (eAppendix, Section 2.2 and eFigure 1 in Supplement 1), overdose history, and fentanyl exposure, which allowed us to capture heterogeneities in overdose risk and naloxone access across population groups and geographic locations. Figure 1 shows the schematic diagram of the model. The microsimulation model runs on a monthly cycle, and we used it to simulate transitions between health states and drug use patterns among the simulated individuals and to project the number of opioid overdose events in each month. We then used the decision tree model to assess the potential pathway and consequence of each overdose event in each month, including setting of overdose^18,19^ (private or semiprivate vs public, see details in Figure 1) and whether the overdose was witnessed, naloxone was administered, emergency medical services were dispatched, emergency department care was used, and the overdose was fatal. In the decision tree, we incorporated a naloxone availability algorithm in which we assumed that the probability of naloxone being available at an overdose event in a city or town was a nonlinear function of the number of naloxone kits in circulation and the number of individuals at risk for opioid overdose in the same jurisdiction. The model accounted for the monthly entry of new naloxone kits into circulation and the exit of existing kits to determine naloxone availability over time. A detailed description for the model is available in the eAppendix, Section 2.0, in Supplement 1 (eTables 1-7 and eFigures 1-2 in Supplement 1).

Because a previous analysis^7^ was built using data before the COVID-19 period (up 2019), we updated our model by incorporating the most recent naloxone distribution data between 2020 and 2022 to increase naloxone availability at baseline.^8^ The updated data included naloxone distributed from OEND programs and pharmacies. We also extend our model calibration to match the annual number of OODs in 2020 and 2021 and to capture the spike in opioid overdoses during the COVID-19 pandemic by adjusting the proportion of fentanyl exposure among people who exclusively used stimulants during the pandemic period (March 2020 and after) (eAppendix, Sections 3.0 and 4.0; eFigures 3-5; and eTables 8-9 in Supplement 1).^8^

### Modeling Scenarios

The status quo scenario represents maintaining the current level of naloxone distribution in 2022, including those from OEND programs (36 694 kits) and pharmacies (15 950 kits),^8^ and the probability of an overdose being witnessed, estimated indirectly from model calibration (0.51 [95% credible interval, 0.34-0.74] in private or semiprivate settings and 0.81 [95% credible interval, 0.76-0.88] in public settings).^20^ The opioid settlement scenario simulated the impact of the state receiving an annual supply of 50 000 intranasal naloxone kits from the opioid settlement for distribution through OEND programs. These 50 000 kits are not entirely additional supplies because they are intended to replace the existing intranasal kits distributed through OEND programs. With the approximately 3000 intramuscular kits already distributed in 2022 through OEND programs accounted for, the total number of naloxone kits available for distribution through OEND programs in this counterfactual scenario was thus 53 000 (16 306 more kits than the status quo scenario). We assumed the level of naloxone kits distributed through pharmacies will remain unchanged. Consistent with a previous study,^7^ we evaluated 2 implementation approaches to distributing additional naloxone kits, aiming to assess whether a demand-oriented (geospatially more equitable) distribution could yield additional benefits: (1) a supply-based approach, for which the distribution of additional naloxone kits is consistent with the historical spatial patterns of naloxone distribution using 2022 data for the proportions of kits received by residents of each city or town; and (2) a demand-based approach, for which the distribution of additional naloxone kits is consistent with the current spatial distribution of individuals at risk for opioid overdose (model estimates), assuming OEND programs could direct the additional kits to new geographic areas if required.

Next, we modeled hypothetical interventions that aim to reduce solitary drug use and thus increase the likelihood of witnessed overdoses (and therefore the opportunity for naloxone to be administered) in private or semiprivate settings. Given the scarcity of evidence on the effectiveness of these relatively new or pilot interventions,^15^ we assessed discrete levels of incremental increases in the probability of an overdose being witnessed in these settings, from 0% (baseline) to 20%, 40%, and 60% (approximately equivalent to the probability of overdoses being witnessed in public settings). We did not model interventions that aim to increase the likelihood of naloxone administration during overdose events occurring in public settings because these settings account for only a few opioid overdoses,^21^ interventions to address public overdoses are distinct from those intended in private or semiprivate settings, and the probability of witnessed overdoses in public settings was already more than 80% in the model.

### Statistical Analysis

We evaluated model outcomes under the various naloxone distribution scenarios (status quo vs opioid settlement) and different probabilities of overdoses being witnessed, both individually and in combination. Results were forecasted for a 3-year time horizon (January 2023 to December 2025), assuming the modeled interventions will be sustained during this period. The primary outcome we examined was the annual number of OODs in 2025 under different scenarios, and we present the results as the mean and 95% simulation interval (SI). In addition, we calculated a ratio of fatal to nonfatal opioid overdoses as a metric for describing improvements in opioid overdose mortality risk (ie, lower ratios indicate reduced fatality risk per opioid overdose). This ratio may offer a more appropriate measure for evaluating the impact of harm reduction services, such as naloxone distribution, because these interventions aim to improve survival (conditional on an overdose occurring) rather than reducing the incidence of overdoses.

To account for the potential increase in solitary drug use during the COVID-19 pandemic period that may have influenced the OODs and uncertainty surrounding the persistence of the pandemic’s impact, we performed sensitivity analysis for 2 scenarios: (1) a scenario in which the probability of overdose witnessing was reduced by 50% from March 2020 onward and (2) a scenario in which the increase in proportion of fentanyl exposure among people who exclusively use stimulants was eliminated from January 2023 onward (eAppendix, Section 5.0 in Supplement 1). The PROFOUND microsimulation model was built with R software, version 4.0.5 (R Foundation for Statistical Computing).^22^

## Results

**Figure 2.**
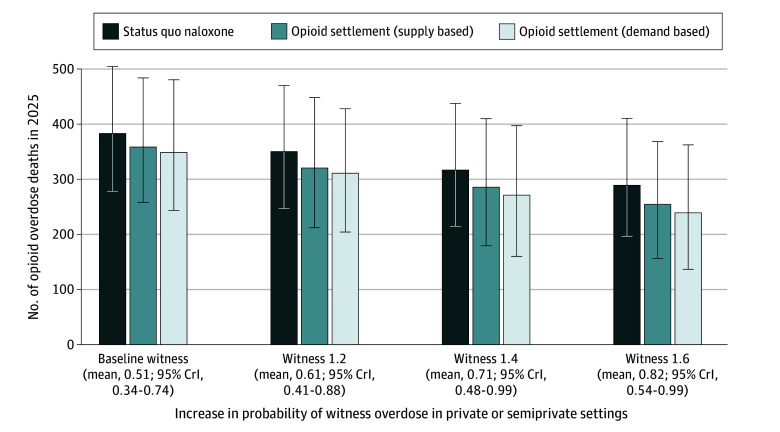
Estimated Number of Opioid Overdose Deaths in 2025 in Different Naloxone Distribution Expansion and Interventions to Increase Probability of Witnessed Overdoses Baseline witness represents the probability of an overdose being witnessed in private or semiprivate settings estimated during model calibration. Levels of witnessed overdoses are represented by the mean and 95% credible interval (CrI) of the calibrated values.

**Figure 3.**
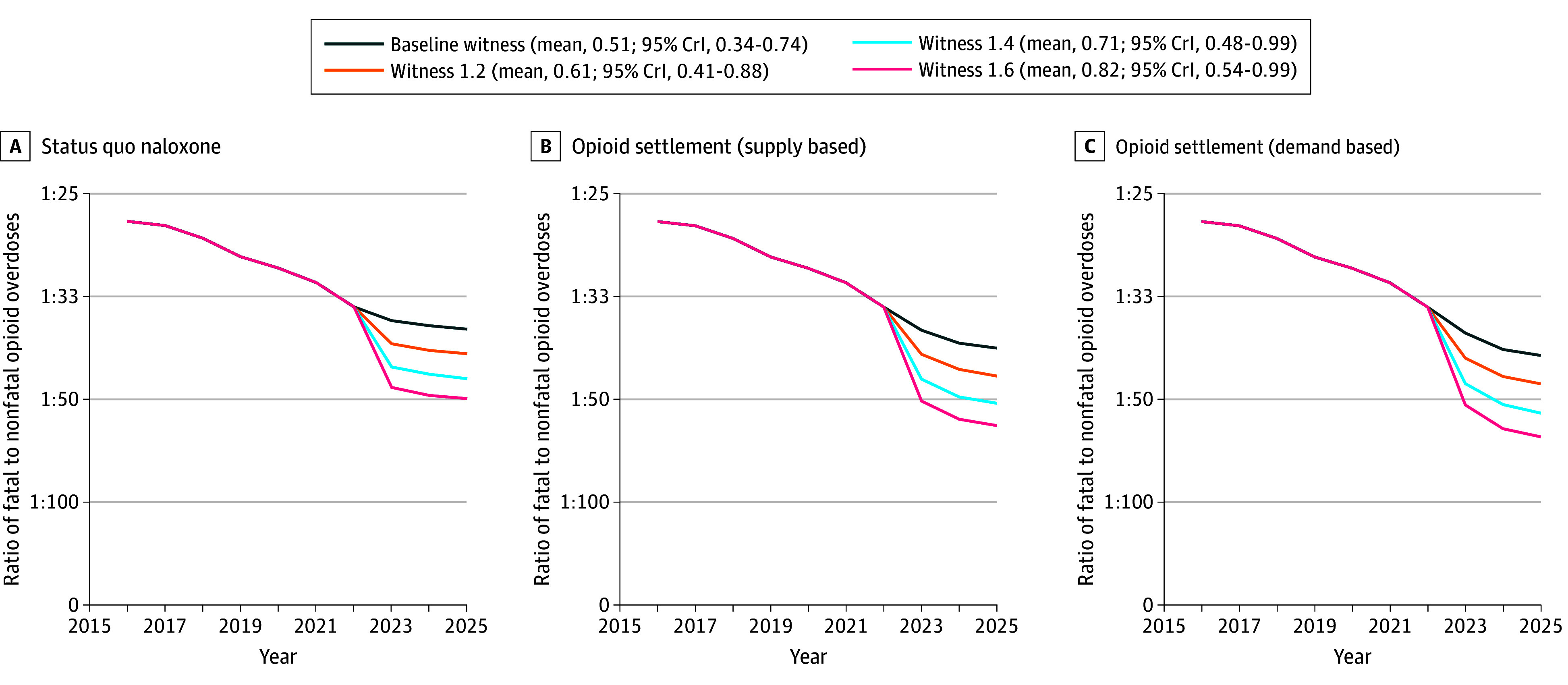
Projected Ratio of Fatal to All Opioid Overdoses in Rhode Island, 2016-2025 Baseline witness represents the probability of an overdose being witnessed in private or semiprivate settings estimated from model calibration. Levels of witnessed overdoses are represented by the mean and 95% credible interval (CrI) of the calibrated values.

After calibration, our model demonstrated excellent fit to the OOD target in 2020 and 2021 (eFigure 4 in Supplement 1). Under the status quo naloxone distribution and assuming a baseline probability of overdoses being witnessed, our model projected 384 (95% SI, 279-507) OODs in 2025 (compared with 378 observed in 2021) (Figure 2; eTable 10 in Supplement 1). We estimated a steady decrease in the ratio of fatal to nonfatal opioid overdoses even without an increase in naloxone availability or probability of witnessed overdoses, from 1:34.5 in 2022 to 1:37.3 in 2025 (Figure 3). This decrease can be attributed to the continuous efforts to expand naloxone distribution between 2016 and 2022 as well as the cumulative increase in the number of naloxone kits in circulation.

Receiving and distributing 53 000 naloxone kits annually (including 50 000 intranasal naloxone kits funded by the opioid settlement) alone could reduce the OODs in 2025 by 24 (95% SI, 1-50; relative reduction, 6.3%; 95% SI, 0.3%-13.7%) and 34 (95% SI, 7-63; relative reduction, 8.8%; 95% SI, 1.8%-17.5%) using the supply-based and demand-based approach, respectively, compared with the status quo scenario (Figure 2). Both distribution approaches resulted in a small decrease in the ratio of fatal to nonfatal opioid overdoses compared with the status quo (1:40.0 and 1:41.2 in 2025, respectively) (Figure 3).

Increasing the probability of witnessed overdoses in private or semiprivate settings has the potential to further reduce the number of OODs without any additional increase in naloxone distribution from the status quo. The potential effect of increasing the probability of witnessed overdoses ranged from 33 deaths averted (95% SI, 0-56; relative reduction, 8.5%; 95% SI, 0.0%-20.3%) if the probability increased by 20% to 93 deaths averted (95% SI, 33-149; relative reduction, 24.1%; 95% SI, 8.6%-39.3%) if the probability increased by 60% in 2025 (Figure 2). With a 60% increase in this probability, the ratio of fatal to nonfatal opioid overdoses decreased to 1:49.9 in 2025 (Figure 3).

We estimated a greater reduction in OODs when combining the expanded naloxone distribution and interventions to increase the probability of witnessed overdoses than the sum of the outcomes of each of these strategies alone. Compared with the status quo naloxone and baseline probability of witnessed overdoses, the combination (with the probability of witnessed overdoses increased by 60%) could avert as many as 129 (95% SI, 63-190; relative reduction, 33.5%; 95% SI, 17.1%-50.4%) and 144 (95% SI, 74-206; relative reduction, 37.4%; 95% SI, 19.6%-56.3%) OODs in 2025 with supply-based and demand-based distribution approaches. In comparison, when implementing the 2 interventions separately, a total of 117 (30.5%) supply-based and 127 (32.5%) demand-based OODs would be averted. Compared with increasing witnessed overdose by 60% alone, the addition of expanded naloxone from opioid settlement increased the reduction in the OODs to 36 (12.3%) and 51 (17.4%) with the 2 distribution approaches, as opposed to 24 (6.3%) and 34 (8.8%) under the baseline probability of witnessed overdoses. Consistent with the previous study,^7^ the demand-based approach yielded fewer OODs compared with the supply-based approach across all scenarios. The lowest ratio of fatal to nonfatal opioid overdoses was found in the combination scenario, with a 60% increase in the probability of witnessed overdoses and a demand-based approach to increased naloxone distribution (1:61.2 in 2025).

In the sensitivity analysis on the reduced probability of witnessed overdoses, we found a decreased impact, in terms of relative reduction in OODs, for expanded naloxone distribution, an increased impact for interventions to increase overdose witnessing, and stronger synergistic outcomes when both were combined. In contrast, the impact remained unchanged in the scenario in which we assumed that the impact of COVID-19 disappeared from January 2023 onward (eFigures 6-7 in Supplement 1).

## Discussion

In this decision analytical model representing all people at risk for opioid overdose in Rhode Island, we found a moderate association of further expansions to community-based naloxone distribution efforts supported by the state’s opioid settlement with future OODs. This finding is likely due to Rhode Island’s robust naloxone distribution program; for example, in 2022 alone, Rhode Island, with a population slightly greater than 1 million, distributed more than 50 000 kits (including those distributed through pharmacies and hospitals), far exceeding the 20 naloxone kits per OOD ratio target level of naloxone distribution previously set,^23^ as well as a saturation point of naloxone needed that was previously calculated for Rhode Island according to a modeling study.^6^ This finding underscores a diminishing marginal impact of additional naloxone kits distributed, aligning with the assumptions underlying our naloxone availability algorithms and a previous modeling work,^6^ and explains the differences in outcomes between supply-based and demand-based distribution methods. Specifically, the demand-based distribution, focusing distribution efforts in areas with lower coverage of naloxone, can lead to a greater increase in the probability of naloxone effectively reversing overdoses for each additional naloxone kit distributed. We found that the impact of further increasing naloxone availability could be enhanced when combined with hypothetical interventions addressing solitary drug use. Furthermore, the ratio of fatal to nonfatal opioid overdoses improved during our study period across all naloxone distribution scenarios (including status quo), although more rapidly when the proportion of witnessed overdoses increased.

One of the major challenges in addressing OODs is the high prevalence of solitary drug use, as individuals who use drugs alone are less likely to receive timely medical intervention (including naloxone administration) when an overdose occurs.^24^ Qualitative studies have suggested several variables associated with solitary drug use, including concerns for withdrawal symptom management, preference for privacy, safety concerns, stigma, not wanting to share drugs with others, and convenience.^25,26^ One cross-sectional study among people attending a substance use disorder treatment program found a significant association among anticipated stigma, polysubstance use, and use in a new setting with using opioids alone.^27^ An analysis of drug overdose death data in Rhode Island from 2016 to 2021 revealed that only 49% of the fatal overdose cases had at least 1 bystander present at the time of the overdose, and among those cases, fewer than half of the bystanders responded to the overdose victims, with some major reasons for not responding being spatial separation, lack of awareness that the individual was using drugs, and failure to recognize the signs of an overdose.^20^

Our study findings highlight the need for interventions that increase the possibility of a bystander witnessing an overdose, along with greater naloxone distribution, to reduce OODs. Recognizing the importance of social and environmental factors that influence settings for drug use, public health efforts may encompass destigmatizing drug use, reducing isolation, housing-focused interventions, education and overdose awareness events, public messaging, peer-to-peer witnessing, and social support to reduce solitary drug use and improve overdose response.^28,29,30^ Additionally, there are several interventions currently being piloted or launched to prevent OODs among people who use drugs alone. For example, OPCs are an evidence-based harm-reduction intervention that provide a space where people can consume drugs under staff supervision so that they can intervene immediately in the event of an overdose. Overdose prevention centers are associated with reduced overdose deaths, substance use–related harms, and transmission of infectious diseases and increased treatment engagement and were found to be cost-effective in a previous study.^14^ The first publicly recognized OPCs in the US are being piloted in Providence, Rhode Island, and New York City; 603 overdose reversals and 0 deaths have been recorded in the first year of operation of OPCs in New York City.^31^ Global innovations in overdose detection technologies could also increase overdose detection and response to reduce OODs.^15^ Brave (an app that anonymously connects people who use drugs alone to help in the event that they overdose), Never Use Alone (a US hotline to support, virtually supervise, and dispatch responders), and the National Overdose Response Service (a similar Canadian hotline) are currently available. Although these emerging overdose detection technologies present unique strengths, such as low cost and improved scalability and reach (particularly in rural areas),^32^ there are some challenges in adopting these technologies, such as low awareness, lack of funding for implementation and evaluation, high costs, and privacy concerns. Although our study is limited by the lack of established estimates regarding the effectiveness of these interventions to address solitary drug use, future research on the effectiveness and safety of these interventions is warranted.

### Limitations

Aside from limitations pertaining to the model itself that have been previously described,^7^ such as no explicit inclusion of status for medications for opioid use disorders and other less common drug use patterns (eg, fentanyl-contaminated benzodiazepines), this study has several additional limitations. First, our model was not able to capture the full complexity of evolving drug use patterns and heterogeneity of overdose risk among people who use drugs, such as the increasing use of xylazine. However, we were able to account for distinct drug use states (eg, exclusive stimulant use and illicit opioid use with or without stimulant use) and key drivers of overdose risk in these groups, including injection drug use, fentanyl exposure, and lifetime history of overdose. Second, given the dearth of other reliable data on the prevalence of stimulant use, we used the prevalence estimates from the National Survey on Drug Use and Health, despite previous research indicating a propensity for National Survey on Drug Use and Health estimates to be underestimated. Third, in contrast to the limited observed surveillance data used as calibration targets, many model parameters were unobservable or have substantial uncertainty, leading to nonidentifiability for a subset of parameters during calibration. However, we believe this nonidentifiability did not compromise the validity of our inferences regarding the effectiveness of 2 interventions under assessment because our model closely matched with the observed target data, the primary determinants of the effectiveness of these interventions. Fourth, in calibrating the model to account for the increase in OODs during the COVID-19 pandemic, we considered only changes to fentanyl exposure among people who exclusively use stimulants. We did not include other potential underlying factors, such as unexpected changes in the unregulated drug supply, interruptions to medications for opioid use disorders and emergency medical services,^33^ and increased solitary drug use,^34^ due to a lack of evidence, evidence that the services were not significantly affected during the pandemic in Rhode Island, or interruptions being temporary.^8^ Although we conducted sensitivity analysis on the potential reduction in overdose witnessing during the COVID-19 pandemic, we aim to incorporate the manifold effects of COVID-19 more comprehensively in future research as more evidence emerges. Fifth, we focused solely on the expansion of naloxone availability in OEND programs from the opioid settlement and did not consider other potential influences in the 2023 to 2025 period, such as the availability of over-the-counter naloxone in pharmacies, changes in the availability of medications for opioid use disorders due to removal of federal restrictions on practitioners (X-waivers), or shifts in the capacity of the harm reduction workforce to sustain community outreach and naloxone distribution efforts. If access to these services is enhanced, our estimates regarding the effectiveness of naloxone distribution and interventions to improve witnessed overdoses may reflect a more optimistic outlook.

## Conclusions

Although increased naloxone provision supported by opioid settlements (and other major sources of funding) can offer significant public health benefits, combining expanded naloxone distribution with efforts to increase witnessed overdoses could result in a greater reduction in OODs at a population level. There are other gaps in the literature that need to be addressed to strengthen future simulation models, including the role of new naloxone formulations, changes in naloxone pricing as a result of over-the-counter status, effectiveness of new overdose prevention interventions, and the impact of targeted naloxone distribution efforts. Because of its focus on a single small state with well-established naloxone distribution programs, this study may not be generalizable to other jurisdictions with lower coverage of naloxone distribution. Nonetheless, as we delved into solitary drug use, a widespread but understudied issue nationally, this study suggests that interventions that increase the likelihood of overdoses being witnessed and naloxone administration may become critical components of a comprehensive strategy to maximize the impact of naloxone distribution and effectively use opioid settlement funds.

## Notes

Supplement 1. **eAppendix.** Supplementary Information **eFigure 1.** Drug Use State Stratification **eFigure 2.** Map of Cities/Towns in Rhode Island **eTable 1.** Past Year Opioid Misuse and Exclusive Stimulant Use Based on the NSDUH Data (Before Adjustment) **eTable 2.** Variable Names Used in the NSDUH Data Derivation **eTable 3.** Study Population Parameters **eTable 4.** Risk of Opioid Overdose for Each Opioid Use Health State **eTable 5.** Monthly Health State Transition Probability Matrix **eTable 6.** Background Age-Stratified Mortality Rate Excluding Death Due to Overdose **eTable 7.** Decision Tree Parameters **eFigure 3.** Results of Preliminary Model Calibration to Observed Fatal and Non-Fatal Overdose Setting Data **eTable 8.** Preliminary Calibration Parameters and Targets **eTable 9.** Calibrated Parameters and Values **eFigure 4.** Model Calibration to Observed Opioid Overdose Deaths in Rhode Island (2016-2021) **eFigure 5.** Model Validation to the City/Town-Level Number of Opioid Overdose Deaths in 2019 **eTable 10.** Model Results for Annual Opioid Overdose Deaths in 2025 Under Different Modeling Scenarios **eFigure 6.** Sensitivity Analysis on the Reduced Probability of Witnessed Overdoses **eFigure 7.** Sensitivity Analysis on the Persistence of the COVID-19 Impacts
eReferences

Supplement 2.
Data Sharing Statement
