# Supplementary material for: Evaluation of Strategies to Enhance Community-Based Naloxone Distribution Supported by an Opioid Settlement
Source: JAMA Netw Open. 2024 May 30;7(5):e2413861. doi: 10.1001/jamanetworkopen.2024.13861 (PMC11140538; doi:10.1001/jamanetworkopen.2024.13861)
Supplement: Supplement 1. — eAppendix. Supplementary Information eFigure 1. Drug Use State Stratification eFigure 2. Map of Cities/Towns in Rhode Island eTable 1. Past Year Opioid Misuse and Exclusive Stimulant Use Based on the NSDUH Data (Before Adjustment) eTable 2. Variable Names Used in the NSDUH Data Derivation eTable 3. Study Population Parameters eTable 4. Risk of Opioid Overdose for Each Opioid Use Health State eTable 5. Monthly Health State Transition Probability Matrix eTable 6. Background Age-Stratified Mortality Rate Excluding Death Due to Overdose eTable 7. Decision Tree Parameters eFigure 3. Results of Preliminary Model Calibration to Observed Fatal and Non-Fatal Overdose Setting Data eTable 8. Preliminary Calibration Parameters and Targets eTable 9. Calibrated Parameters and Values eFigure 4. Model Calibration to Observed Opioid Overdose Deaths in Rhode Island (2016-2021) eFigure 5. Model Validation to the City/Town-Level Number of Opioid Overdose Deaths in 2019 eTable 10. Model Results for Annual Opioid Overdose Deaths in 2025 Under Different Modeling Scenarios eFigure 6. Sensitivity Analysis on the Reduced Probability of Witnessed Overdoses eFigure 7. Sensitivity Analysis on the Persistence of the COVID-19 Impacts eReferences [file jamanetwopen-e2413861-s001.pdf]

Supplementary Online Content

Zang X, Skinner A, Krieger MX, et al. Evaluation of strategies to enhance community-based naloxone distribution supported by an opioid settlement. *JAMA Netw Open*. 2024;7(5):e2413861. doi:10.1001/jamanetworkopen.2024.13861

eAppendix. Supplementary Information
eFigure 1. Drug Use State Stratification
eFigure 2. Map of Cities/Towns in Rhode Island
eTable 1. Past Year Opioid Misuse and Exclusive Stimulant Use Based on the NSDUH Data (Before Adjustment)
eTable 2. Variable Names Used in the NSDUH Data Derivation
eTable 3. Study Population Parameters
eTable 4. Risk of Opioid Overdose for Each Opioid Use Health State
eTable 5. Monthly Health State Transition Probability Matrix
eTable 6. Background Age-Stratified Mortality Rate Excluding Death Due to Overdose
eTable 7. Decision Tree Parameters
eFigure 3. Results of Preliminary Model Calibration to Observed Fatal and Non-Fatal Overdose Setting Data
eTable 8. Preliminary Calibration Parameters and Targets
eTable 9. Calibrated Parameters and Values
eFigure 4. Model Calibration to Observed Opioid Overdose Deaths in Rhode Island (2016-2021)
eFigure 5. Model validation to the City/Town-Level Number of Opioid Overdose Deaths in 2019
eTable 10. Model Results for Annual Opioid Overdose Deaths in 2025 Under Different Modeling Scenarios
eFigure 6. Sensitivity analysis on the Reduced Probability of Witnessed Overdoses
eFigure 7. Sensitivity Analysis on the Persistence of the COVID-19 Impacts
eReferences

This supplementary material has been provided by the authors to give readers additional information about their work.

eAppendix. Supplementary Information

1.0 Overview

The **P**revention and **R**escue **of** **F**entanyl and Other Opioid **O**verdoses **U**sing Optimized **N**aloxone **D**istribution Strategies (PROFOUND) model is an open-source, individual-based microsimulation model designed to enhance the effectiveness of overdose prevention programs by optimizing naloxone distribution strategies. The PROFOUND model simulates the populations of people who are at risk of opioid overdose residing in each geospatial subregion within a given jurisdiction to evaluate the population-level impact and costs of different naloxone distribution strategies. The model provides evidence and outcomes to guide policy makers in choosing the overdose prevention strategies that maximize the use of scarce financial and organizational resources to save lives. Model parameters and inputs are adaptable to different jurisdictions. The model is intended to be tool for decision makers seeking to use available overdose surveillance data to set program priorities for overdose fatality prevention and rescue, while maintaining flexibility to respond to temporal and geographic changes in the opioid overdose burden. Our model code is publicly available at https://github.com/pph-collective/profound-model. To facilitate the use of the model in resource allocation decisions for naloxone distribution, we also developed a webtool to help disseminate the model, visualize model outcomes, and allow users to explore results by choosing modeling scenarios and varying a few key model parameters. This webtool is available at https://profoundmodel.org/ (access to the code is available upon request). In the current study, we aimed to compare alternative strategies to increase naloxone distribution through community-based programs in Rhode Island.

© 2024 Zang X et al. *JAMA Network Open.*

2.0 Model description

2.1 Model structure overview

The PROFOUND model is an individual-based model composed of a microsimulation and a decision tree model. The individual-based microsimulation allows the inclusion of stochastic variation as well as variation due to individual characteristics, capturing population heterogeneities.[1] It also overcomes the Markovian assumption required by Markov models by allowing for "memory" in the model's structure, such as the individual's history of overdose. The microsimulation model is used to track changes in health and drug use states and to forecast overdose events among simulated individuals. The decision tree is used to determine the potential pathway and consequence of each overdose event.

2.2 Study population

We initialized the simulated study population representing all individuals in Rhode Island age 12 years and older who are at risk for opioid overdose, including people with exclusive prescription opioid misuse (excluding those who also use illicit opioids), any non-injection illicit opioid use (excluding those who also inject illicit opioids), any illicit opioid use via injection, and people who exclusively use stimulants without intended opioid use (but whose substances may be contaminated by fentanyl, see eFigure 1). We also included people with an opioid use history but who are currently not actively using opioids. In addition, we characterized the simulated study population by sex (male/female), age (continuous), race/ethnicity (Black/African American [Black], Hispanic/Latino [Hispanic], non-Hispanic white [White], and other [Other]), city/town of residence (39 in total), overdose history (yes/no), and fentanyl exposure (yes/no).

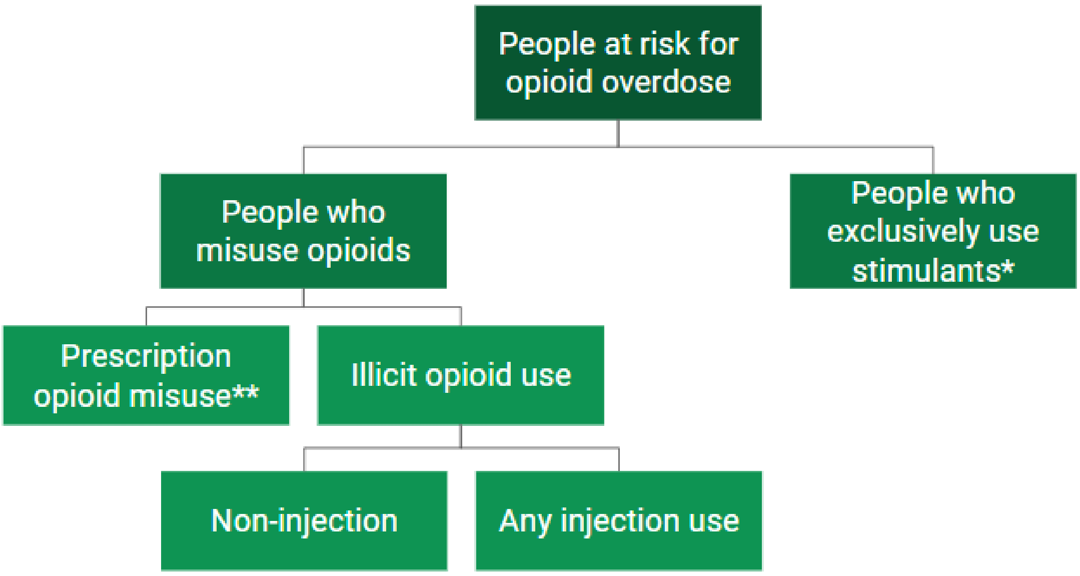

eFigure 1. Drug Use State Stratification

* Whose drugs may be contaminated with fentanyl; ** exclusive prescription opioid misuse.

In this study, we defined city/town as the subregion in Rhode Island (eFigure 2). In the study population initialization for the microsimulation, we first determined the

© 2024 Zang X et al. *JAMA Network Open.*

population size and size of each demographic group (race * age group * sex) in each city/town based on the American Community Survey (2010 Census) for Rhode Island.[2] Using the National Survey on Drug Use and Health (NSDUH) data (northeast region),[3] we also estimated the prevalence of opioid misuse (all types) and exclusive stimulant use (cocaine and methamphetamine) among each demographic group. We then combined results from these two datasets to estimate the opioid misuse populations within each demographic group in each city/town. We compared the resulting estimated total number of people with opioid misuse with the Rhode Island's overall estimate, based on a statewide assessment of the prevalence of opioid use disorder for Rhode Island (5.2%)[4] and the state population, and adjusted the demographic and jurisdiction-specific estimates by a multiplier that reflects the difference between the two statewide assessments. We determined the population who exclusively use stimulants (without intended opioid use) within each demographic group in each city/town using the NSDUH data without adjustment (eTable 1).

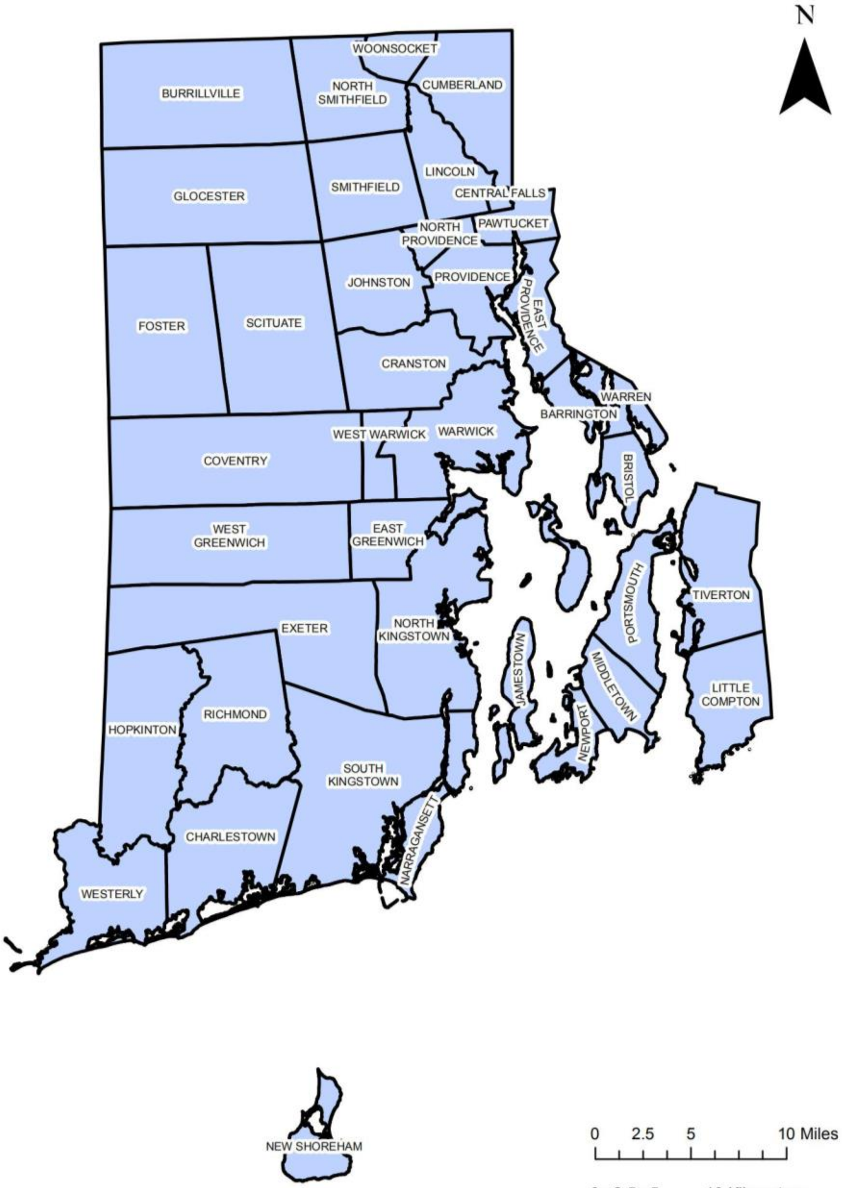

© 2024 Zang X et al. *JAMA Network Open.*

eFigure 2. Map of Cities/Towns in Rhode Island

We then used the NSDUH dataset to derive, among the people with opioid misuse of different sex, the proportion of each type of opioid use. The variable names we used in the estimation process with the NSDUH data are presented in eTable 2. We also used a statewide cross-sectional assessment of the cascade of care for opioid use disorder in Rhode Island[4] to determine the proportion of the simulated people with an opioid use history but who are currently not actively using opioids (defined as in recovery from opioid use disorder).

eTable 1. Past Year Opioid Misuse and Exclusive Stimulant Use Based on the NSDUH Data (Before Adjustment)

Sex	Race/ethnicity	Age group	Past year opioid misuse, percentage	Past year stimulant use, percentage
Male	White	12 to 17	0.0249	0.0039
Male	White	18 to 25	0.0671	0.0561
Male	White	26 to 34	0.0700	0.0485
Male	White	35 to 49	0.0570	0.0224
Male	White	50 to 64	0.0377	0.0128
Male	White	≥ 65	0.0159	0.0022
Male	Black	12 to 17	0.0261	0.0011
Male	Black	18 to 25	0.0537	0.0166
Male	Black	26 to 34	0.0689	0.0203
Male	Black	35 to 49	0.0414	0.0190
Male	Black	50 to 64	0.0376	0.0376
Male	Black	≥ 65	0.0251	0.0068
Male	Hispanic	12 to 17	0.0244	0.0034
Male	Hispanic	18 to 25	0.0629	0.0409
Male	Hispanic	26 to 34	0.0592	0.0394
Male	Hispanic	35 to 49	0.0460	0.0258
Male	Hispanic	50 to 64	0.0267	0.0120
Male	Hispanic	≥ 65	0.0291	0.0030
Male	Other	12 to 17	0.0222	0.0038
Male	Other	18 to 25	0.0556	0.0453
Male	Other	26 to 34	0.0603	0.0377
Male	Other	35 to 49	0.0418	0.0259
Male	Other	50 to 64	0.0301	0.0152
Male	Other	≥ 65	0.0180	0.0058
Female	White	12 to 17	0.0277	0.0034
Female	White	18 to 25	0.0675	0.0392
Female	White	26 to 34	0.0595	0.0274
Female	White	35 to 49	0.0437	0.0103
Female	White	50 to 64	0.0313	0.0063
Female	White	≥ 65	0.0117	0.0010
Female	Black	12 to 17	0.0352	0.0015

Female	Black	18 to 25	0.0463	0.0121
Female	Black	26 to 34	0.0447	0.0109
Female	Black	35 to 49	0.0308	0.0122
Female	Black	50 to 64	0.0313	0.0146
Female	Black	≥ 65	0.0083	0.0000
Female	Hispanic	12 to 17	0.0361	0.0038
Female	Hispanic	18 to 25	0.0524	0.0317
Female	Hispanic	26 to 34	0.0478	0.0229
Female	Hispanic	35 to 49	0.0307	0.0114
Female	Hispanic	50 to 64	0.0132	0.0051
Female	Hispanic	≥ 65	0.0209	0.0000
Female	Other	12 to 17	0.0303	0.0046
Female	Other	18 to 25	0.0609	0.0322
Female	Other	26 to 34	0.0478	0.0174
Female	Other	35 to 49	0.0299	0.0100
Female	Other	50 to 64	0.0240	0.0051
Female	Other	≥ 65	0.0139	0.0000

eTable 2. Variable Names Used in the NSDUH Data Derivation

Variable	Variable name	Definition in NSDUH	Estimation
Opioid misuse	HERPNRYR	HEROIN USE AND/OR PAIN RELIEVER MISUSE - PAST YEAR	
Illicit opioid use	HERYR	HEROIN - PAST YEAR USE	
Prescription opioid misuse			(HERPNRYR = 1) – (HERYR = 1)
Illicit opioid use (Injection)	HRNDLREC	TIME SINCE LAST USED NEEDLE TO INJECT HEROIN (within in the past 12 months)	
Illicit opioid use (Injection)			(HERYR = 1) – (HRNDLREC = 1)
Stimulant use (without opioid use)	COCYR<br>METHAMYR	COCAINE - PAST YEAR USE<br>METHAMPHETAMINE - PAST YEAR USE	(COCYR = 1) OR (METHAMYR = 1) – (HERPNRYR = 1)

We then assigned the initial overdose history variable value (yes/no) to simulated study individuals, stratified by type of drug use based on evidence from literature (eTable 3). This variable is updated during the micro-simulation when overdoses occur. The initial level of fentanyl exposure (intentional use of fentanyl or fentanyl-contaminated drugs) was also estimated from the literature.[5-8] We assumed that fentanyl exposure is limited to people who use illicit opioids, stimulants, and prescription opioids not from prescribers, whereas people who exclusively misuse prescription opioids sourced from prescribers (including those from friends/relatives who were prescribed these opioids) are not at risk for fentanyl exposure. Estimates for the source of prescription opioids

© 2024 Zang X et al. *JAMA Network Open.*

were based on NSDUH data. We also applied a monthly increase of 0.5% [0% - 1%][9] in the proportion of individuals exposed to fentanyl (same growth rate applied to people who use illicit opioids, stimulants, and prescription opioids not from prescribers) to account for secular trends in the period before March 2020. eTable 3 presents the resulting list of parameters used in defining the study population.

eTable 3. Study Population Parameters

Parameter	Value	Range	Source
Population size in Rhode Island	1,059,000		2
Prevalence of opioid misuse	5.2%	3.7% – 6.7%	4
Proportion of illicit opioid use among males who misuse opioids	16.6%		NSDUH
Proportion of illicit opioid use among females who misuse opioids	12.1%		NSDUH
Proportion of illicit opioid use (via injection) among males who use illicit opioids	48.6%		NSDUH
Proportion of illicit opioid use (via injection) among females who use illicit opioids	41.1%		NSDUH
Proportion of stimulant use (via injection) among people who exclusively use stimulants	13.3%		3
Proportion of inactive opioid use status among people who misuse opioids	9.1%	8.6% – 9.6%	4
Proportion of people with prescription opioid misuse who have ever overdosed	19.1%		10
Proportion of people with illicit opioid use (non-injection) who have ever overdosed	25.2%		11
Proportion of people with illicit opioid use (injection) who have ever overdosed	47.9%		12
Proportion of people with stimulant use (without opioid use) who have ever overdosed	25.2%		11
Proportion of prescription opioids from sources other than from doctor	19.23%		NSDUH
Initial proportion of fentanyl exposure among people who use non-prescription opioids	56.4%	41.6% - 86.6%	5-7
Initial proportion of fentanyl exposure among people who exclusively use stimulants	15.7%	14.3% - 17.0%	8
Monthly growth in fentanyl exposure	0.5%	0 – 1%	9

2.3 Opioid overdose risk (microsimulation)

Naloxone is only effective in reversing overdoses caused by opioids. We modeled opioid overdose only and assumed different monthly risks of overdose for different patterns of opioid use based on the literature (eTable 4). Overdose events in each monthly time-step are randomly drawn among simulated individuals according to these probabilities of experiencing overdose. In general, people who use illicit opioids face a higher risk of overdose than those with exclusive prescription opioid misuse and those

© 2024 Zang X et al. *JAMA Network Open.*

who use injection illicit opioids have a higher risk of overdose than those who use non-injection illicit opioids. We accounted for several factors that can elevate risk of overdose: (1) fentanyl exposure; (2) overdose history, where risk for subsequent overdose was assumed to be higher than risk for the initial overdose; and (3) first month of opioid use after being in the inactive opioid use state (see Section 2.4 below), to account for elevated risk associated with decreased tolerance for opioids after a period of abstinence. We assumed that the risk of opioid overdose was zero for people in the inactive opioid use state and people who exclusively use stimulants but whose drugs are not contaminated by fentanyl. In the absence of clear evidence about the risk of opioid overdose among those whose stimulants were contaminated by fentanyl, we assumed this risk was equal to the risk for those whose non-injection illicit opioids contain fentanyl.

Simulated individuals who experience opioid overdose in each monthly time-step are assigned subsequent outcomes using the decision tree (see Section 2.5 below).

eTable 4. Risk of Opioid Overdose for Each Opioid Use Health State

Parameter	Value	Range	Source
Monthly probability of overdose (subsequent), exclusive prescription opioid misuse	0.0026	0.00121 – 0.00455	[13]
Monthly probability of overdose (subsequent), non-injection illicit opioid use	0.0154	0.0122 – 0.0186	[14]
Relative risk of overdose for injection opioid use (compared to non-injection illicit opioid use)	3.1	1.8 – 4.4	[15,16]
Relative risk of overdose for fentanyl exposure	6.07	3.63 – 10.16	[17,18]
Relative risk of overdose during the first month of opioid relapse	4.3	3.6 – 5.2	[19]
Relative risk of overdose for subsequent versus first overdose	3.5	1.9 – 6.4	[14]

2.4 Transitions between health states (microsimulation)

Individuals' transitions between health states are determined using a random process drawn from a transition probability matrix (eTable 5). Transition probabilities were estimated from the published literature.

If a simulated individual is in the exclusive prescription opioid misuse health state, in each monthly time-step this individual is subject to a monthly probability of transiting to non-injecting illicit opioid use;[20] individuals in the non-injection illicit opioid use health state can transition to illicit injection opioid use;[21] and individuals in the illicit injection opioid use health state can transition back to illicit non-injection opioid use[22]. Individuals in each of these three opioid use states can also transition between these

© 2024 Zang X et al. *JAMA Network Open.*

opioid use states and an inactive opioid use state (due to treatment or recovery).[23-25] We also included as a separate health state for the first month of opioid use after being in the inactive state (relapse)[26] to account for the elevated risk of overdose, after which the simulated individuals would transit (with a transition probability of 1) to the same active opioid use state prior to being in the inactive opioid use state. For simplicity, we did not account for potential transitions between opioid use states and stimulant use states, and we did not include inactive stimulant use state.

In addition, simulated individuals in all health states are subject to age-specific risk of death due to causes other than overdose ("background mortality"), which we calculated by converting published annual death rates (based on life tables[27]) into monthly probabilities (eTable 6) and applying a multiplier to reflect higher risks among people who use drugs.[28]

eTable 5. Monthly Health State Transition Probability Matrix

From ***To***	**Prescription opioid**	**Non-injection illicit opioid**	**Injection illicit opioid**	**Inactive opioid use**
**Prescription opioid**	-	0	0	0
**Illicit non-injection opioid**	0.000418	-	0.0148	0
**Illicit injection opioid**	0	0.00824	-	0
**Inactive***	0.00374	0.00595	0.00254	-
**Relapse (1 month)**	0	0	0	0.0452

***Data presented here represent the estimates for the initial period, while the transition probabilities into the inactive opioid use period are subject to a monthly growth rate of 0.59% according to the increase in the number of people on medication assisted treatment in Rhode Island.[29]

eTable 6. Background Age-Stratified Mortality Rate Excluding Death Due to Overdose

**Age group**	**All-cause death rate among general population, per 100, 000**		**Death rate among people who use drugs, per 100, 000***	
	**Annual**	**Monthly**	**Annual**	**Monthly**
14 years and under	13.3	1.11	34.4	2.87
15 to 24 years	70.2	5.85	181.8	15.2
25 to 34 years	128.8	10.7	333.6	27.8
35 to 44 years	194.7	16.2	504.3	42.1
45 to 54 years	359.9	30.0	932.1	78.0
55 to 64 years	886.7	74.2	2296.6	193.4
65 years and over	4386.1	373.1	11360.0	999.9

* Calculated using a multiplier for the mortality rate among people who use drugs compared to general population. Only the death rates among people who use drugs were used in the model.

© 2024 Zang X et al. *JAMA Network Open.*

2.5 Opioid overdose events (decision tree)

We used a decision tree module to determine the pathways and consequences for each overdose event. Model parameters for the decision tree are presented in eTable 7. In the decision tree, each node is associated with branches defined by a set of probabilities that add up to one. The probabilities vary depending on the characteristics of the simulated individuals and the outcomes from the previous nodes. These nodes include the setting of the overdose (public versus private/semi-private), whether the overdose is witnessed, whether naloxone is administered (among witnessed overdoses), whether emergency medical services (EMS) is called (among witnessed overdoses), whether the overdosed individual receives emergency department (ED) care (among those for whom EMS is called), and whether the individual dies or survives.

In the decision tree, the probabilities of an overdose being witnessed and of a witness calling for help from EMS depends on the setting of overdose (public versus private/semi-private). The probability of dying from an overdose depends on whether naloxone is administered and whether EMS is called. The baseline probability of dying from an overdose where no naloxone is administered nor EMS called is based on observational studies.[30,31] Unlike previous modeling studies, we did not assume the probability of dying when naloxone is administered is zero; instead we derived this estimate from a systematic review of observational studies which used a pooled estimate of the proportion of opioid overdoses where naloxone kits were used that resulted in death.[32] This may be a conservative assumption about the effectiveness of naloxone but it may also be warranted in the current era of fentanyl use. We also included a lower relative risk of death from overdose when EMS is called but no naloxone is administered by a witness.[33,34]

If the simulated individual survives, this individual then returns to the microsimulation either in the inactive opioid use health state[35] or in the same active drug use health state as prior to overdose, but with a history of overdose.

eTable 7. Decision Tree Parameters

Parameter	Value	Range*	Source
Proportion of overdoses occurring in public settings	0.12	0.05 – 0.31	Assumption
Probability of an overdose being witnessed in public settings	0.82	0.75 – 0.88	17,18
Relative risk of an overdose being witnessed in private/semi-private versus public settings	0.6	0.2 – 1	Assumption
Probability of witness(es) calling EMS in public settings	0.66	0.56 – 0.80	19,36-38
Relative risk of witness(es) calling EMS in private/semi-private versus public settings	0.59	0.4 – 0.7	36,38
Probability of transport to hospital for ED care	0.9	0.85 – 0.95	14

© 2024 Zang X et al. *JAMA Network Open.*

Probability of ceasing opioid use after surviving an overdose	0.076	0.048 – 0.116	[35]
Probability of dying from an overdose (no naloxone, no EMS)	0.0588	0.0310 – 0.108	[30,31]
Probability of dying from an overdose with naloxone administered	0.0086	0.0056 – 0.0132	[30,32]
Relative risk of death from overdose with EMS called (no naloxone)	0.588	0.419 – 0.758	[33,34]

* For parameters entering calibration, the ranges represent prior ranges before calibration

2.6 Naloxone availability algorithm

To estimate the probability of naloxone being available and administered during a witnessed overdose, we adopted a previously used approach by Irvine et al.[39] assuming the probability is a nonlinear function of the number of naloxone kits in circulation and the size of the population at risk for opioid overdose. These variables can vary by city/town:

$$P_t^r = c(1 - \exp\left(-(\tau/c)\frac{NXc_t^r + NXp_t^r * R_{eff}^{pc}}{NP_t^r}\right)$$

$$NXc_t^r = NXc_{t-1}^r(1 - r_w) + NXc_{yr}^r/12$$

$$NXp_t^r = NXp_{t-1}^r(1 - r_w) + NXp_{yr}^r/12$$

where P_t^r denotes the probability that naloxone is available and administered during a witnessed overdose in region *r* at time *t*; c denotes the cap on naloxone availability, which was assumed to be 0.99; τ denotes the adjustment factor for naloxone availability, whose value is determined in model calibration (see Section 3.2); NXc_t^r denotes the number of naloxone kits from OEND (community-based) programs in circulation in region r at time t; NXp_t^r denotes the number of naloxone kits from pharmacies in circulation in region r at time t; R_{eff}^{pc} denotes the ratio of effectiveness of pharmacy programs in reaching at-risk population compared to OEND programs (0.371 [0.345, 0.4], based on an observational study for the correlates of naloxone recipient characteristics with distribution program characterisics[40]); NP_t^r denotes the number of at-risk (for opioid overdose) individuals residing in region r at time t based on model estimates; r_w denotes the monthly rate of naloxone withdrawn due to expiration/loss (1/15.5 months, i.e., 6.5%), based on an unpublished analysis of the New York City Department of Health and Mental Hygiene Naloxone Recipient Form data for the average circulation time for naloxone kits; NXc_{yr}^r denotes the number of naloxone kits from OEND (community-based) programs received by residents in region r in a given year; NXp_t^r denotes the number of naloxone kits from pharmacies received by residents in region r in a given year. Naloxone data for the number of naloxone kits distributed by OEND programs and received by residents of each subregion (i.e., city/town) were derived from data collected by the Rhode Island Department of Health (RIDOH) using a statewide, standardized reporting form for each

individual receiving naloxone kit(s) from any OEND programs. Because subregional level data for naloxone distribution from pharmacies were unavailable in Rhode Island, we used the aggregate annual number of naloxone kits distributed by pharmacies in the state and assumed the amount received was proportional to the size of at-risk population in each subregion.

2.7 Population dynamics

When simulated individuals leave the model due to age-stratified background mortality (eTable 6) or overdose death (eTable 7), the deceased individuals were replaced by new ones with the same initial characteristics except for overdose history (reset as 0). This allowed us to maintain a fixed size of the simulated population of people at risk of overdose in Rhode Island. For simplicity we did not include in-migration/out-migration at the state or city/town level.

© 2024 Zang X et al. *JAMA Network Open.*

3.0 Model calibration

Model calibration refers to the process of matching model outcomes with observed data by adjusting uncertain model parameters and establishing plausible ranges that provide the best fit to available data.[41] Calibrating model inputs to observed epidemiological endpoints ensures the credibility of model results and thus strengthens our confidence in model inferences. We used a two-step calibration procedure for the PROFOUND model. First, we conducted an initial calibration of a smaller set of model parameters associated with overdose setting that are only used in the decision tree model. We then conducted a formal calibration for the entire model.

3.1 Initial calibration to determine overdose setting parameters in the decision tree

During data collection to inform our model, we identified substantial differences between the settings of fatal opioid overdoses in data from the State Unintentional Drug Overdose Reporting Surveillance[42] and Rhode Island Office of the State Medical Examiners[43] compared to data on the settings of non-fatal opioid overdose collected by the Rhode Island Emergency Medical Services (EMS) Information System.[44] In the first source approximately 10% of fatal opioid overdoses were reported to occur in public settings, as compared to approximately 31% of non-fatal opioid overdose reported in EMS data. This difference is likely attributable to the overrepresentation of opioid overdoses occurring in public captured by the EMS system and/or higher survival rates from overdoses occurring in public as a result of higher likelihood of being intervened and rescued. To address these differences, we performed calibration for three parameters: (1) proportion of overdoses occurring in public settings; (2) relative risk of overdose being witnessed in private/semi-private versus public settings; and (3) relative risk of witness(es) calling EMS in private/semi-private versus public settings. The calibration was compared against the two targets for opioid overdose setting derived from fatal overdose surveillance data and EMS data (for non-fatal overdoses). The prior values and distributions for the calibration parameters and targets are presented in eTable 8.

In this initial calibration, we used a Bayesian method with the incremental mixture importance sampling (IMIS) algorithm[45] (using R package IMIS[46]). Given the challenges in calibrating a stochastic model (due to Monte Carlo error),[45] we first transformed the decision tree model from individual-based to cohort-based (i.e., from stochastic to deterministic) assuming a cohort of 10,000 individuals experiencing overdose. All other decision tree parameters were held fixed at their prior point estimates, since they were independently defined from the calibration targets and had low influence on the calibration targets.[45] We generated a posterior sample of one million parameter sets from the IMIS algorithm. The results, including the posterior distribution of calibrated parameters and model fit to calibration targets, are presented in eFigure 2.

© 2024 Zang X et al. *JAMA Network Open.*

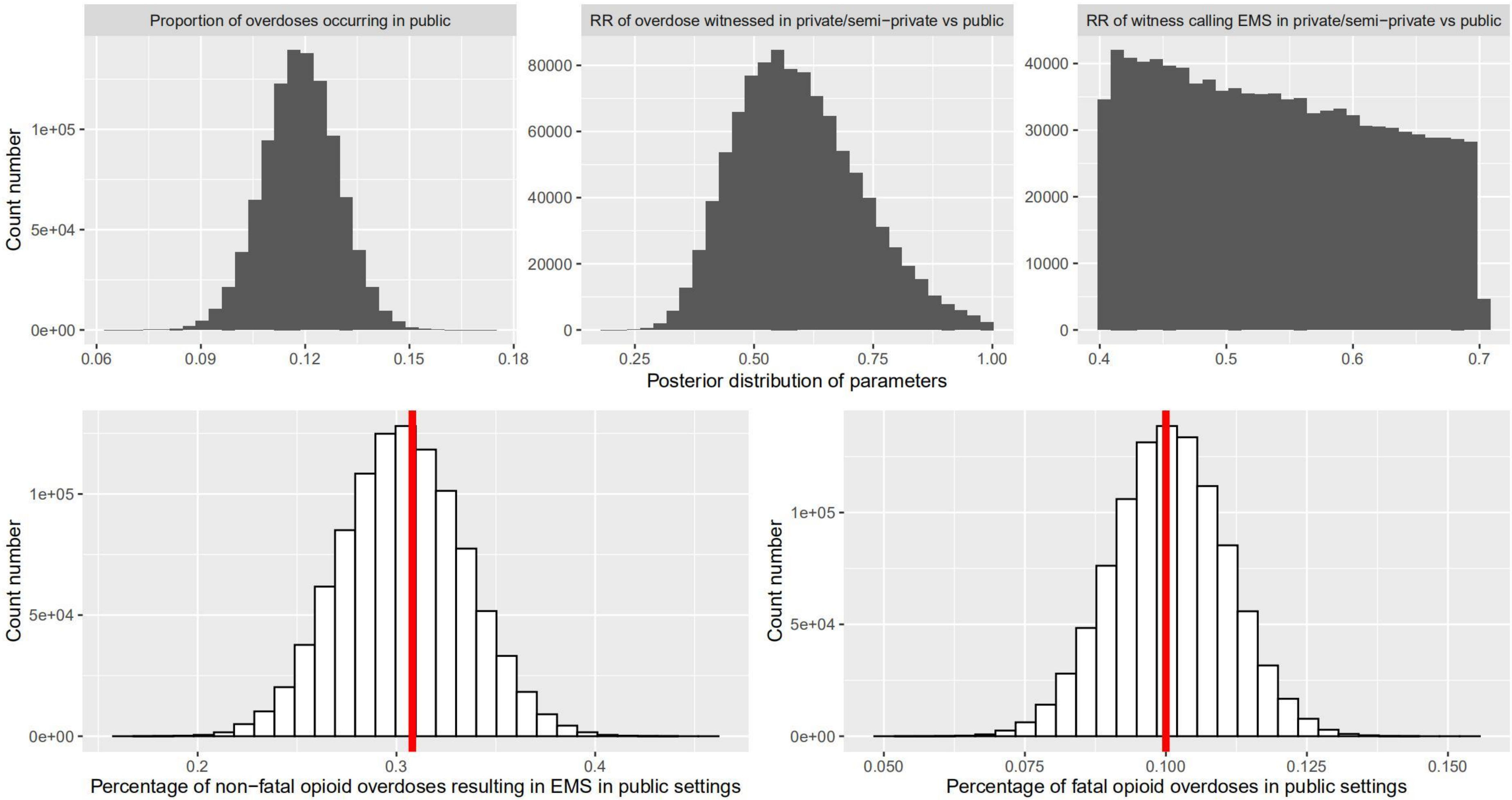

eFigure 3. Results of Preliminary Model Calibration to Observed Fatal and Non-Fatal Overdose Setting Data

RR: relative risk

© 2024 Zang X et al. *JAMA Network Open.*

eTable 8. Preliminary Calibration Parameters and Targets

	Prior value	Prior distribution	Source
**Model parameter**			
Proportion of overdoses occurring in public settings	0.12	Uniform (0.05, 0.31)	Assumption
Relative risk of an overdose being witnessed in private/semi-private versus public settings	0.6	Uniform (0.2, 1)	Assumption
Relative risk of witness(es) calling EMS in private/semi-private versus public settings	0.59	Uniform (0.4, 0.7)	[36]
**Calibration target***			
Percentage of non-fatal opioid overdoses resulting in EMS in public settings	30.8%	Normal (0.308, 0.0314)	[42,43]
Percentage of fatal opioid overdoses in public settings	10%	Normal (0.1, 0.0102)	[44]

3.2 Formal calibration of the entire model before 2020

In the formal calibration for the entire model before 2020, we used a random search calibration approach to repeatedly sample from estimated uncertainty ranges for 16 key model parameters that had the greatest influence on target estimates (eTable 9). We compared model projections against three targets in each year between 2016 to 2019 in Rhode Island reported by the Rhode Island Department of Health: (1) the number of opioid overdose deaths (OODs); (2) the percentage of OODs involving fentanyl; and (3) the number of emergency department visits related to opioid overdose (eTable 9). We used a Latin hypercube sampling method[47] to draw one million random parameters sets from the parameters' uncertainty range, augmented them with the one million sets of overdose setting parameters from the initial calibration and one million random seeds (to ensure reproducibility of results), simulated the model with these sets of inputs and seeds, and compared the resulting model projections against the selected calibration targets. We identified 500 calibrated subsets (and seeds) providing the best goodness-of-fit statistics for subsequent analysis. The goodness-of-fit (GoF) was measured by the mean percentage deviation, as shown in the equation below:

$$GoF = \frac{1}{N}\sum_{i}\frac{|proj_i - obs_i|}{obs_i}$$

where N represents the number of calibration targets, $proj_i$ represents the model-projected result for the ith target, and obs_i represents the observed estimate for the ith target. Smaller values of the GoF indicate a better fit to the observed data.

This approach, together with the stochastic process embedded within the microsimulation design, allowed us to derive calibrated model parameters providing

© 2024 Zang X et al. *JAMA Network Open.*

good fit to observed targets while simultaneously exploring the uncertainty of model outcomes resulting from both parameter uncertainty (the uncertainty in estimation of the parameter of interest) and stochastic uncertainty (random variability in outcomes between identical simulated agents).[41] We present in eTable 9 the posterior value of parameters after this calibration.

eTable 9. Calibrated Parameters and Values

Parameters	Table	Prior value	Prior range	Posterior range (95% credible interval)
Baseline prevalence of fentanyl in opioids from non-prescription sources	eTable 3	56.4%	41.6% – 72.4%	56.8% (42.3% – 71.5%)
Monthly growth in fentanyl prevalence	eTable 3	0.5%	0 – 1%	0.82% (0.44% – 1%)
Monthly probability of subsequent overdose, prescription opioid use	eTable 4	0.0026	0.00121 – 0.00455	0.0024 (0.0013 – 0.0040)
Monthly probability of subsequent overdose, non-injection illicit opioid use	eTable 4	0.0154	0.0122 – 0.0186	0.0154 (0.0123 – 0.0184)
Relative risk of overdose for injection opioid use	eTable 4	3.1	1.8 – 4.4	3.07 (1.85 – 4.33)
Relative risk of overdose for fentanyl exposure	eTable 4	6.07	3.63 – 10.16	6.50 (3.94 – 9.77)
Relative risk of overdose during the first month of opioid relapse	eTable 4	4.3	3.6 – 5.2	4.42 (3.66 – 5.17)
Relative risk of overdose for first versus subsequent overdose	eTable 4	3.5	1.9 – 6.4	4.39 (2.24 – 6.30)
Probability of dying from an overdose (no naloxone, no EMS)	eTable 7	0.0588	0.0310 – 0.108	0.040 (0.032 – 0.053)
Probability of dying from an overdose with naloxone administered	eTable 7	0.0086	0.0056 – 0.0132	0.010 (0.0058 – 0.013)
Relative risk of death from overdose with EMS called (no naloxone)	eTable 7	0.588	0.419 – 0.758	0.58 (0.43 – 0.75)
Probability of an overdose being witnessed in public settings	eTable 7	0.82	0.75 – 0.88	0.81 (0.76 – 0.88)
Probability of witness(es) calling EMS in public settings	eTable 7	0.66	0.56 – 0.80	0.69 (0.57 – 0.79)
Probability of transport to hospital for ED care	eTable 7	0.9	0.85 – 0.95	0.90 (0.85 – 0.95)
Probability of ceasing opioid use after surviving an overdose	eTable 7	0.076	0.048 – 0.116	0.080 (0.050 – 0.114)
Adjustment factor for naloxone availability	Assumed	1	0.75 – 1.25	1.00 (0.77 – 1.23)

© 2024 Zang X et al. *JAMA Network Open.*

3.3 Calibration of model after 2020

Incorporating the most recent naloxone distribution data between 2020 to 2022,[48] we updated our model by calibrating it to match the observed annual number of OODs in 2020 and 2021 to capture the spike in OODs during the COVID-19 pandemic.[9] The surveillance data showed that the annual number of drug overdose deaths involving cocaine increased by nearly 50% from 2019 to 2021. Using the same 500 sets of calibrated parameters from the previous model calibration before 2020, we iteratively adjusted a multiplier to the proportion of fentanyl exposure among people who exclusively use stimulants from March 2020 onwards (using the parameter value from December 2019 as a basis). We assumed a prior range for this multiplier to be 1 to 2, and we adjusted this multiplier until the model projected number of OODs in 2020 and 2021 matched with the observed numbers. Through calibration, we determined this multiplier to be 1.52. Please see eFigure 4 for calibration results. Subsequently, we maintained this parameter at the newly calibrated value throughout our scenario evaluation period (2022 to 2025).

© 2024 Zang X et al. *JAMA Network Open.*

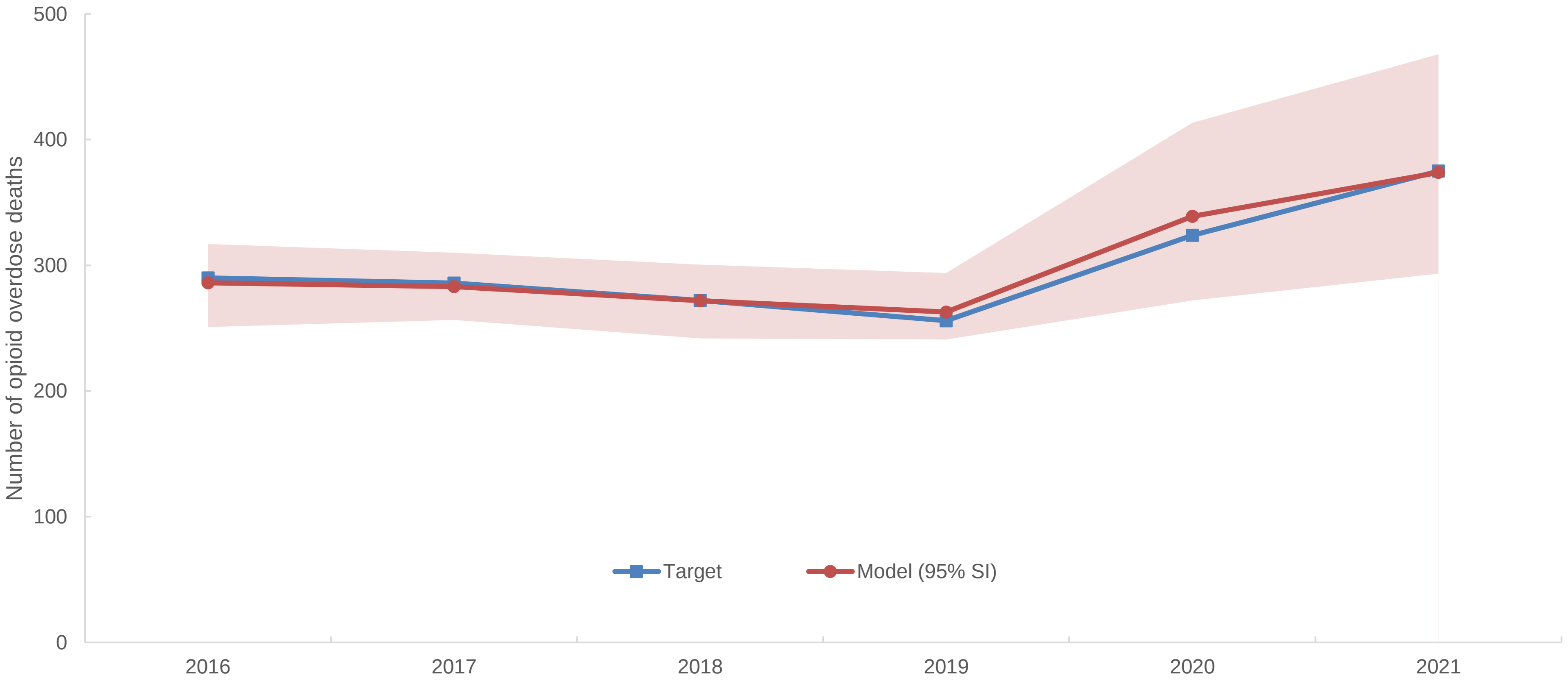

eFigure 4. Model Calibration to Observed Opioid Overdose Deaths in Rhode Island (2016-2021)

SI: simulation interval

© 2024 Zang X et al. *JAMA Network Open.*

4.0 Model validation

Model validation refers to the process of evaluating a model's accuracy in making relevant projections.[49] In particular, external validation entails the comparison of model projections to external estimates of key clinical and epidemiological data not used in the model. Given that our model was calibrated to epidemiological targets at the state level but our model was built to replicate the opioid overdose epidemics in each city/town, we compared our projections for the annual number of OODs in each city/town from the 500 calibrated parameter sets with Rhode Island Department of Health surveillance data at the city/town level from 2019[50] to examine whether the observed numbers fall within the 95% simulation intervals of the model estimates in each jurisdiction (eFigure 5). The results show that the surveillance data for all cities/towns but one (Lincoln) fell within the 95% simulation intervals of the model estimates.

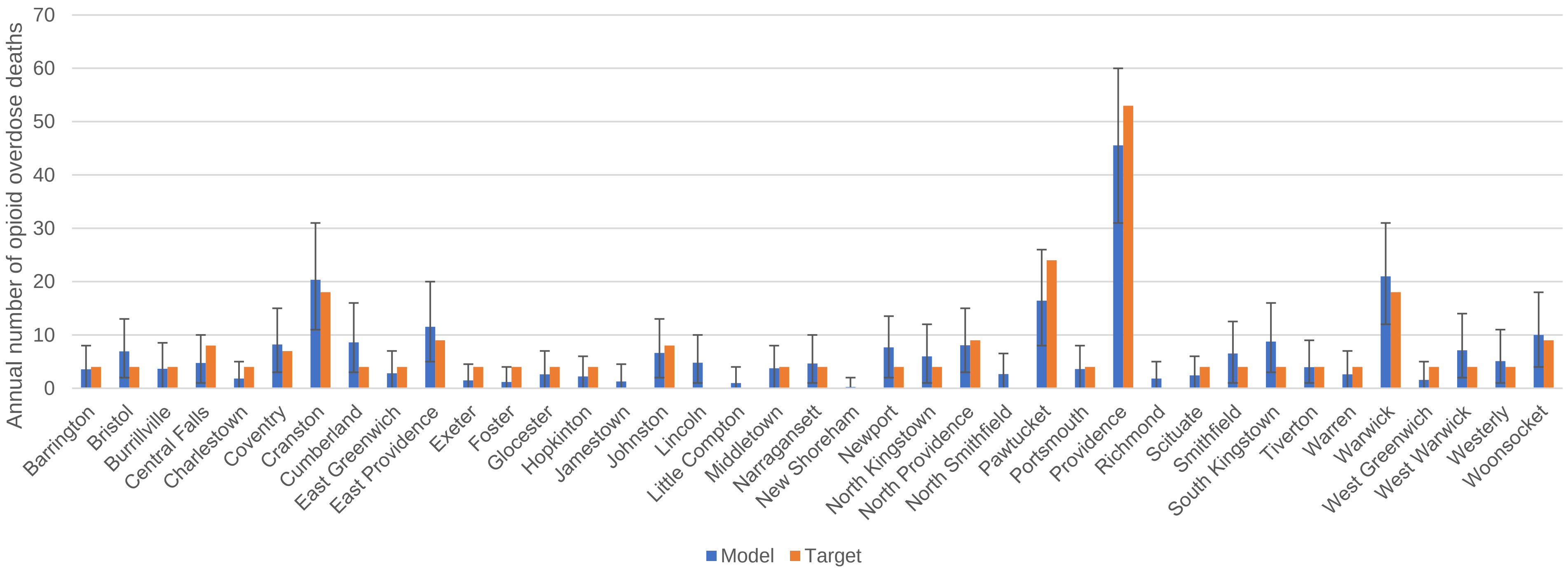

eFigure 5. Model validation to the City/Town-Level Number of Opioid Overdose Deaths in 2019

Legend: suppressed numbers for cities/towns with <5 OODs (required by RIDOH) were recoded as 4 for visualizations purposes and to match the state-level total

© 2024 Zang X et al. *JAMA Network Open.*

eTable 10. Model Results for Annual Opioid Overdose Deaths in 2025 Under Different Modeling Scenarios

Overdose witness in private/semi-private settings	Status quo naloxone	Opioid settlement, supply-based	Opioid settlement, Demand-based
Baseline witness	384 [279, 507]	360 (-6.3%) [260, 486]	351 (-8.8%) [246, 483]
Witness * 1.1	367 (-4.5%) [363, 488]	341 (-11.4%) [235, 461]	331 (-13.9%) [222, 452]
Witness * 1.2	352 (-8.5%) [247, 472]	323 (-16.0%) [213, 450]	313 (-18.7%) [205, 430]
Witness * 1.3	337 (-12.3%) [231, 460]	305 (-20.6%) [197, 431]	294 (-23.6%) [176, 422]
Witness * 1.4	319 (-17.1%) [216, 440]	286 (-25.5%) [180, 411]	273 (-28.9%) [161, 399]
Witness * 1.5	305 (-20.7%) [201, 427]	270 (-29.9%) [168, 394]	256 (-33.4%) [151, 379]
Witness * 1.6	292 (-24.1%) [196, 412]	256 (-33.5%) [157, 370]	241 (-37.4%) [137, 365]

The results are presented by the mean value (relative reduction compared to status quo naloxone with baseline overdose witness) and [95% simulated interval] based on the 500 calibrated model parameter sets.

© 2024 Zang X et al. *JAMA Network Open.*

5.0 Sensitivity analysis

To account for the potential increase in solitary drug use during the COVID-19 pandemic period that may have influenced the opioid overdose deaths and uncertainty surrounding the persistence of the pandemic's impact, we also performed sensitivity analysis for two scenarios: (1) a scenario where the probability of overdose witnessing was reduced by 50% from March 2020 onward **(eFigure 6)**; (2) a scenario where the increase in proportion of fentanyl exposure among people who exclusively use stimulants was eliminated from January 2023 onward **(eFigure 7)**.

© 2024 Zang X et al. *JAMA Network Open.*

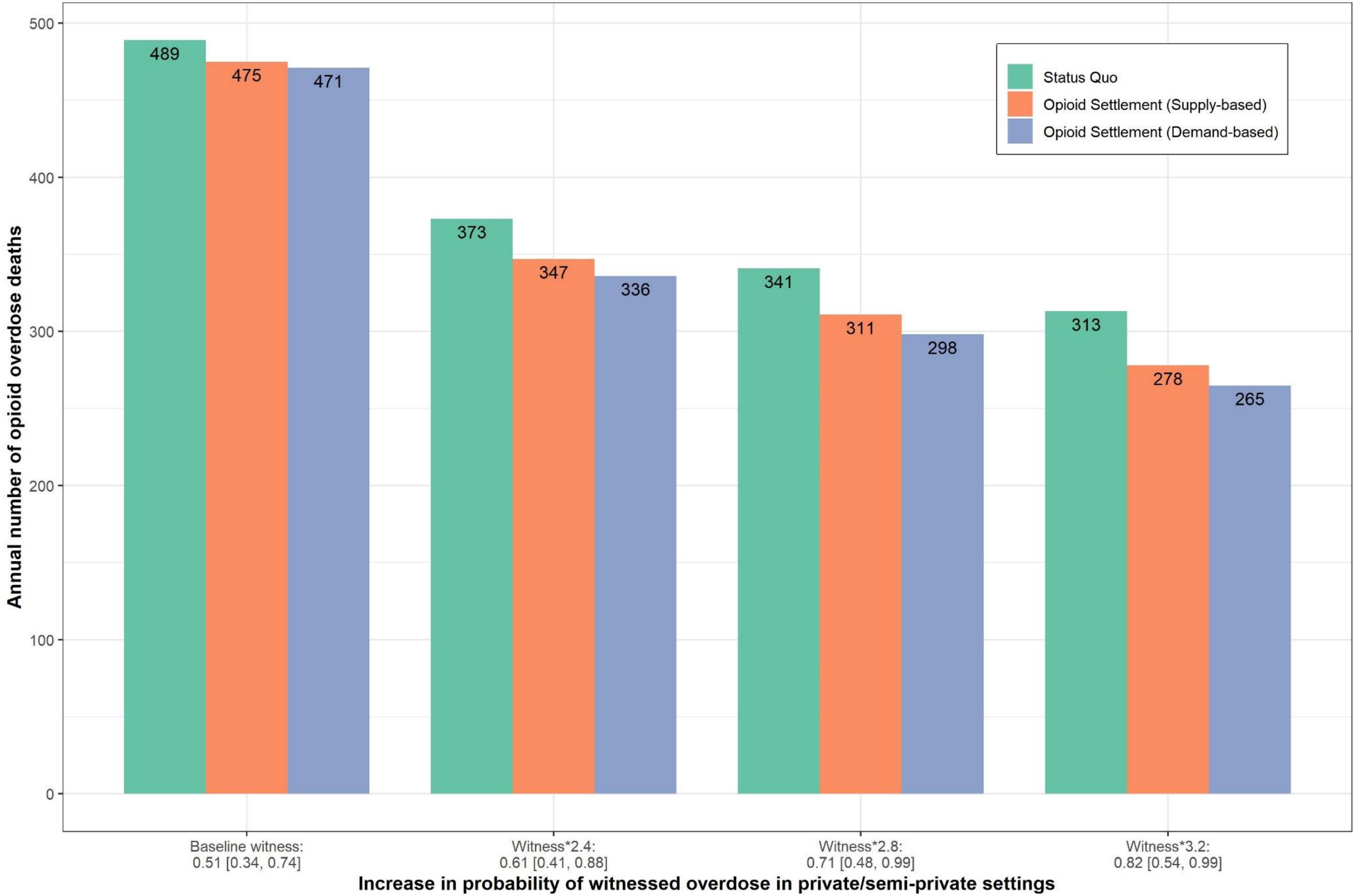

eFigure 6. Sensitivity analysis on the Reduced Probability of Witnessed Overdoses

© 2024 Zang X et al. *JAMA Network Open.*

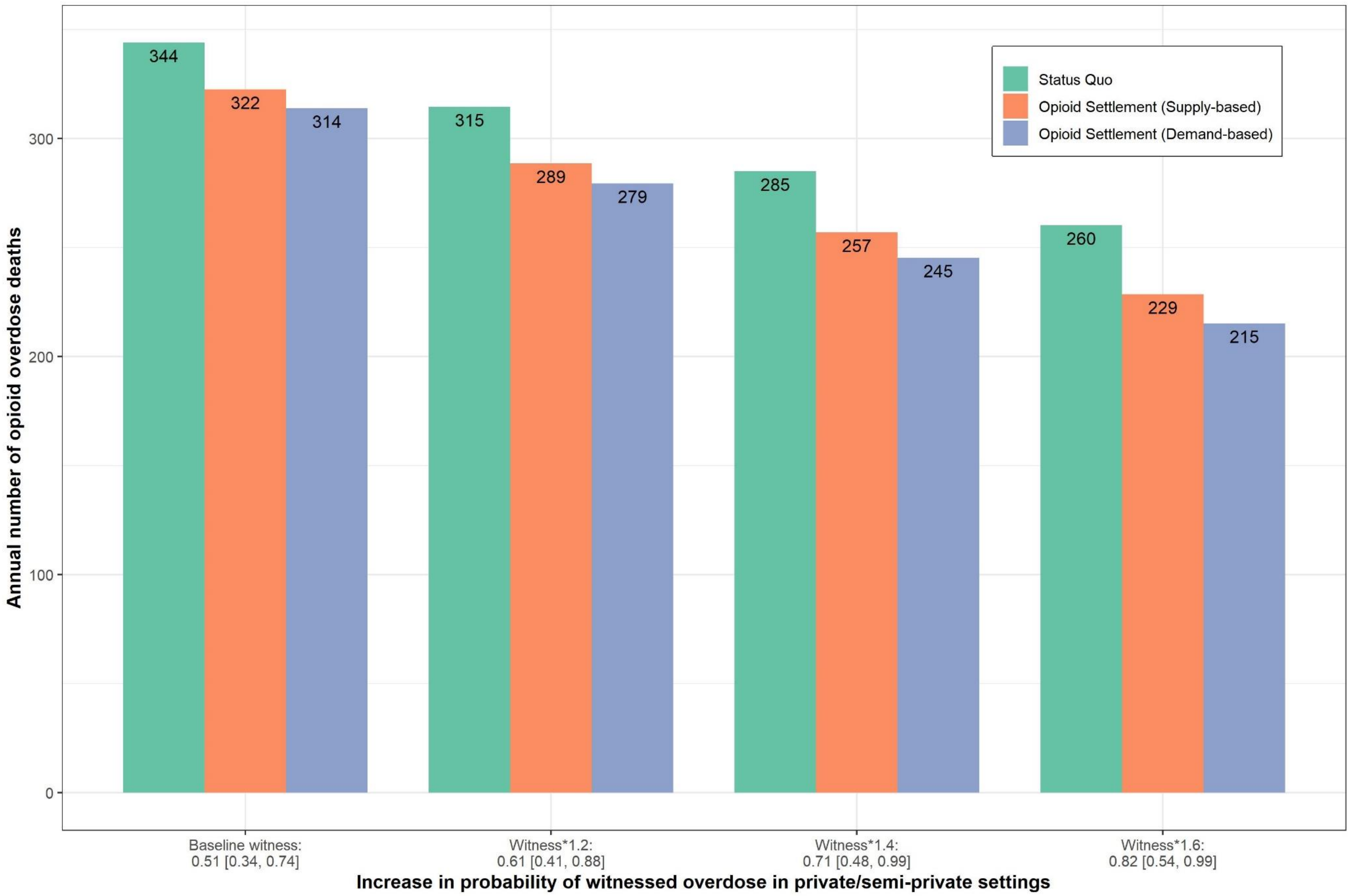

eFigure 7. Sensitivity Analysis on the Persistence of the COVID-19 Impacts

© 2024 Zang X et al. *JAMA Network Open.*

eReferences

1. Krijkamp EM, Alarid-Escudero F, Enns EA, Jalal HJ, Hunink MM, Pechlivanoglou P. Microsimulation modeling for health decision sciences using R: a tutorial. *Medical Decision Making.* 2018;38(3):400-422.
2. Social Explorer Tables (SE), Census 2010, Census Bureau. https://www.socialexplorer.com/explore-tables. Accessed September 9, 2020.
3. Substance Abuse and Mental Health Services Administration. National Survey on Drug Use and Health: 2-Year RDSA (2018-2019). https://rdas.samhsa.gov/#/. Accessed March 5, 2021.
4. Yedinak JL, Goedel WC, Paull K, et al. Defining a recovery-oriented cascade of care for opioid use disorder: a community-driven, statewide cross-sectional assessment. *PLoS medicine.* 2019;16(11):e1002963.
5. Dezman ZD, Felemban W, Bontempo LJ, Wish ED. Evidence of fentanyl use is common and frequently missed in a cross-sectional study of emergency department patients in Baltimore, Maryland. *Clinical Toxicology.* 2020;58(1):59-61.
6. Kenney SR, Anderson BJ, Conti MT, Bailey GL, Stein MD. Expected and actual fentanyl exposure among persons seeking opioid withdrawal management. *Journal of substance abuse treatment.* 2018;86:65-69.
7. Carroll JJ, Marshall BD, Rich JD, Green TC. Exposure to fentanyl-contaminated heroin and overdose risk among illicit opioid users in Rhode Island: A mixed methods study. *International Journal of Drug Policy.* 2017;46:136-145.
8. LaRue L, Twillman RK, Dawson E, et al. Rate of fentanyl positivity among urine drug test results positive for cocaine or methamphetamine. *JAMA Network Open.* 2019;2(4):e192851-e192851.
9. Prevent Overdose RI. Overdose Death Data, Rhode Island. https://preventoverdoseri.org/overdose-deaths/. Accessed May 20, 2023.
10. Dunn KE, Barrett FS, Fingerhood M, Bigelow GE. Opioid overdose history, risk behaviors, and knowledge in patients taking prescribed opioids for chronic pain. *Pain medicine.* 2017;18(8):1505-1515.
11. Martins SS, Sampson L, Cerdá M, Galea S. Worldwide prevalence and trends in unintentional drug overdose: a systematic review of the literature. *American journal of public health.* 2015;105(11):e29-e49.
12. Seal KH, Kral AH, Gee L, et al. Predictors and prevention of nonfatal overdose among street-recruited injection heroin users in the San Francisco Bay Area, 1998–1999. *American Journal of Public Health.* 2001;91(11):1842-1846.
13. Dunn KM, Saunders KW, Rutter CM, et al. Opioid prescriptions for chronic pain and overdose: a cohort study. *Annals of internal medicine.* 2010;152(2):85-92.
14. Darke S, Williamson A, Ross J, Mills KL, Havard A, Teesson M. Patterns of nonfatal heroin overdose over a 3-year period: findings from the Australian treatment outcome study. *Journal of Urban Health.* 2007;84(2):283-291.
15. Kerr T, Fairbairn N, Tyndall M, et al. Predictors of non-fatal overdose among a cohort of polysubstance-using injection drug users. *Drug and alcohol*

© 2024 Zang X et al. *JAMA Network Open.*

dependence. 2007;87(1):39-45.

16. Brugal MT, Barrio G, Fuente LDL, Regidor E, Royuela L, Suelves JM. Factors associated with non-fatal heroin overdose: assessing the effect of frequency and route of heroin administration. *Addiction.* 2002;97(3):319-327.
17. Latimer J, Ling S, Flaherty I, Jauncey M, Salmon AM. Risk of fentanyl overdose among clients of the Sydney Medically Supervised Injecting Centre. *International journal on drug policy.* 2016;37:111-114.
18. Otachi JK, Vundi N, Surratt HL. Examining factors associated with non-fatal overdose among people who inject drugs in rural appalachia. *Substance Use & Misuse.* 2020;55(12):1935-1942.
19. Merrall EL, Kariminia A, Binswanger IA, et al. Meta-analysis of drug-related deaths soon after release from prison. *Addiction.* 2010;105(9):1545-1554.
20. Banerjee G, Edelman EJ, Barry DT, et al. High-dose prescribed opioids are associated with increased risk of heroin use among US military veterans. *Pain.* 2019;160(9):2126.
21. Neaigus A, Gyarmathy VA, Miller M, Frajzyngier VM, Friedman SR, Des Jarlais DC. Transitions to injecting drug use among noninjecting heroin users: social network influence and individual susceptibility. *JAIDS Journal of Acquired Immune Deficiency Syndromes.* 2006;41(4):493-503.
22. Reddon H, DeBeck K, Socias ME, et al. Frequent cannabis use and cessation of injection of opioids, Vancouver, Canada, 2005–2018. *American journal of public health.* 2020;110(10):1553-1560.
23. Scherrer JF, Salas J, Sullivan MD, et al. Impact of adherence to antidepressants on long-term prescription opioid use cessation. *The British Journal of Psychiatry.* 2018;212(2):103-111.
24. Huo D, Bailey SL, Ouellet LJ. Cessation of injection drug use and change in injection frequency: the Chicago Needle Exchange Evaluation Study. *Addiction.* 2006;101(11):1606-1613.
25. Nosyk B, Li L, Evans E, et al. Characterizing longitudinal health state transitions among heroin, cocaine, and methamphetamine users. *Drug and alcohol dependence.* 2014;140:69-77.
26. Evans JL, Hahn JA, Lum PJ, Stein ES, Page K. Predictors of injection drug use cessation and relapse in a prospective cohort of young injection drug users in San Francisco, CA (UFO Study). *Drug and alcohol dependence.* 2009;101(3):152-157.
27. Murphy SL, Xu J, Kochanek KD, Arias E, Tejada-Vera B. Final data for 2018. National Vital Statistics Reports. *Hyattsville, MD: National Center for Health Statistics.* 2020;vol 69, no 13.
28. Lindblad R, Hu L, Oden N, Wakim P, Rosa C, VanVeldhuisen P. Mortality rates among substance use disorder participants in clinical trials: pooled analysis of twenty-two clinical trials within the National Drug Abuse Treatment Clinical Trials Network. *Journal of substance abuse treatment.* 2016;70:73-80.
29. Prevent Overdose, RI. Medication-Assisted Treatment Data, Rhode Island. https://preventoverdoseri.org/medication-assisted-therapy/. Accessed May 4,

© 2024 Zang X et al. *JAMA Network Open.*

2021.
30. Bird SM, Parmar MK, Strang J. Take-home naloxone to prevent fatalities from opiate-overdose: protocol for Scotland's public health policy evaluation, and a new measure to assess impact. *Drugs: education, prevention and policy.* 2015;22(1):66-76.
31. Giglio RE, Li G, DiMaggio CJ. Effectiveness of bystander naloxone administration and overdose education programs: a meta-analysis. *Injury epidemiology.* 2015;2(1):1-9.
32. McDonald R, Strang J. Are take‐home naloxone programmes effective? Systematic review utilizing application of the Bradford Hill criteria. *Addiction.* 2016;111(7):1177-1187.
33. Townsend T, Blostein F, Doan T, Madson-Olson S, Galecki P, Hutton DW. Cost-effectiveness analysis of alternative naloxone distribution strategies: first responder and lay distribution in the United States. *International Journal of Drug Policy.* 2020;75:102536.
34. Sumner SA, Mercado-Crespo MC, Spelke MB, et al. Use of naloxone by emergency medical services during opioid drug overdose resuscitation efforts. *Prehospital Emergency Care.* 2016;20(2):220-225.
35. Langabeer J, Champagne-Langabeer T, Luber SD, et al. Outreach to people who survive opioid overdose: Linkage and retention in treatment. *Journal of Substance Abuse Treatment.* 2020;111:11-15.
36. Karamouzian M, Kuo M, Crabtree A, Buxton JA. Correlates of seeking emergency medical help in the event of an overdose in British Columbia, Canada: findings from the Take Home Naloxone program. *International Journal of Drug Policy.* 2019;71:157-163.
37. Ambrose G, Amlani A, Buxton JA. Predictors of seeking emergency medical help during overdose events in a provincial naloxone distribution programme: a retrospective analysis. *BMJ open.* 2016;6(6):e011224.
38. Lim JK, Forman LS, Ruiz S, et al. Factors associated with help seeking by community responders trained in overdose prevention and naloxone administration in Massachusetts. *Drug and alcohol dependence.* 2019;204:107531.
39. Irvine MA, Oller D, Boggis J, et al. Estimating naloxone need in the USA across fentanyl, heroin, and prescription opioid epidemics: a modelling study. *The Lancet Public Health.* 2022;7(3):e210-e218.
40. Moustaqim-Barrette A, Papamihali K, Mamdani Z, Williams S, Buxton JA. Accessing Take-Home Naloxone in British Columbia and the role of community pharmacies: Results from the analysis of administrative data. *Plos one.* 2020;15(9):e0238618.
41. Briggs AH, Weinstein MC, Fenwick EA, et al. Model parameter estimation and uncertainty: a report of the ISPOR-SMDM Modeling Good Research Practices Task Force--6. *Value in health : the journal of the International Society for Pharmacoeconomics and Outcomes Research.* 2012;15(6):835-842.
42. James McDonald M, Goldschmidt A, Koziol J, McCormick M, Viner-Brown S,

© 2024 Zang X et al. *JAMA Network Open.*

Alexander-Scott N. State unintentional drug overdose reporting surveillance: opioid overdose deaths and characteristics in Rhode Island. *Rhode Island Medical Journal.* 2018;101(7):25-30.

43. Weidele HR, Scagos RP. Accidental drug overdose deaths in Rhode Island: January 1, 2016–July 31, 2020. *Rhode Island Medical Journal.* 2020;103(10):62-65.
44. Lasher L, Jason Rhodes MPA A-C, Viner-Brown S. Identification and description of non-fatal opioid overdoses using Rhode Island EMS data, 2016–2018. *Rhode Island Medical Journal.* 2019;102(2):41-45.
45. Menzies NA, Soeteman DI, Pandya A, Kim JJ. Bayesian methods for calibrating health policy models: a tutorial. *Pharmacoeconomics.* 2017;35(6):613-624.
46. Raftery AE, Bao L. Estimating and projecting trends in HIV/AIDS generalized epidemics using incremental mixture importance sampling. *Biometrics.* 2010;66(4):1162-1173.
47. Helton JC, Davis FJ. Latin hypercube sampling and the propagation of uncertainty in analyses of complex systems. *Reliability Engineering & System Safety.* 2003;81(1):23-69.
48. Prevent Overdose RI. Naloxone Data, Rhode Island. https://preventoverdoseri.org/naloxone-data/. Accessed March 26, 2023.
49. Eddy DM, Hollingworth W, Caro JJ, Tsevat J, McDonald KM, Wong JB. Model transparency and validation: a report of the ISPOR-SMDM Modeling Good Research Practices Task Force–7. *Medical Decision Making.* 2012;32(5):733-743.
50. PreventOverdoseRI. Overdose Death Data, Rhode Island. https://preventoverdoseri.org/overdose-deaths/. Accessed October 18, 2020.

© 2024 Zang X et al. *JAMA Network Open.*
