# Supplementary material for: Evaluation of Strategies to Enhance Community-Based Naloxone Distribution Supported by an Opioid Settlement
Source: JAMA Netw Open. 2024 May 30;7(5):e2413861. doi: 10.1001/jamanetworkopen.2024.13861 (PMC11140538; doi:10.1001/jamanetworkopen.2024.13861)
Supplement: Supplement 2. — Data Sharing Statement [file jamanetwopen-e2413861-s002.pdf]

Data Sharing Statement

Zang. Evaluation of Strategies to Enhance Community-Based Naloxone Distribution Supported by an Opioid Settlement. *JAMA Netw Open*. Published May 30, 2024.
doi:10.1001/jamanetworkopen.2024.13861

Data
Data available: Yes
Data types: Data (not involving human participants)
How to access data: Data will be made available in the manuscript and its appendix
When available: With publication

Supporting Documents
Document types: None

Additional Information
Who can access the data: anyone requesting the data
Types of analyses: for any purpose
Mechanisms of data availability: with investigator support
